# Structural and Functional Studies of *H*. *seropedicae* RecA Protein – Insights into the Polymerization of RecA Protein as Nucleoprotein Filament

**DOI:** 10.1371/journal.pone.0159871

**Published:** 2016-07-22

**Authors:** Wellington C. Leite, Carolina W. Galvão, Sérgio C. Saab, Jorge Iulek, Rafael M. Etto, Maria B. R. Steffens, Sindhu Chitteni-Pattu, Tyler Stanage, James L. Keck, Michael M. Cox

**Affiliations:** 1 Department of Physics, Ponta Grossa State University (UEPG), Av. Carlos Cavalcanti, 4748, CEP. 84.030–900, Ponta Grossa, PR, Brazil; 2 Department of Structural and Molecular Biology and Genetics, Ponta Grossa State University (UEPG), CEP 84030–900, Ponta Grossa, PR, Brazil; 3 Department of Chemistry, Ponta Grossa State University (UEPG), CEP 84030–900, Ponta Grossa, PR, Brazil; 4 Department of Biochemistry and Molecular Biology, Federal University of Parana, CEP 81531–980 Curitiba, Brazil; 5 Department of Biochemistry, University of Wisconsin–Madison, Madison, WI, 53706–1544, United States of America; 6 Department of Biomolecular Chemistry, University of Wisconsin School of Medicine and Public Health, Madison, WI, 53706, United States of America; University of Iowa, UNITED STATES

## Abstract

The bacterial RecA protein plays a role in the complex system of DNA damage repair. Here, we report the functional and structural characterization of the *Herbaspirillum seropedicae* RecA protein (HsRecA). HsRecA protein is more efficient at displacing SSB protein from ssDNA than *Escherichia coli* RecA protein. HsRecA also promotes DNA strand exchange more efficiently. The three dimensional structure of HsRecA-ADP/ATP complex has been solved to 1.7 Å resolution. HsRecA protein contains a small N-terminal domain, a central core ATPase domain and a large C-terminal domain, that are similar to homologous bacterial RecA proteins. Comparative structural analysis showed that the N-terminal polymerization motif of archaeal and eukaryotic RecA family proteins are also present in bacterial RecAs. Reconstruction of electrostatic potential from the hexameric structure of HsRecA-ADP/ATP revealed a high positive charge along the inner side, where ssDNA is bound inside the filament. The properties of this surface may explain the greater capacity of HsRecA protein to bind ssDNA, forming a contiguous nucleoprotein filament, displace SSB and promote DNA exchange relative to EcRecA. Our functional and structural analyses provide insight into the molecular mechanisms of polymerization of bacterial RecA as a helical nucleoprotein filament.

## Introduction

The bacterial RecA protein plays a role in the complex system of DNA damage repair. RecA protein catalyzes strand exchange reaction between single-strand DNA (ssDNA) and homologous double-strand DNA (dsDNA) molecules, and also induces the expression of DNA repair proteins in response to DNA damage through a regulatory network known as the SOS response [1–8].

The *Escherichia coli* RecA protein (EcRecA) monomers bind onto DNA producing a right-handed helical nucleoprotein filament [9,10]. Recently the EcRecA crystal structures of RecA-ssDNA and RecA-dsDNA complexes have been reported [11], confirming that each RecA monomer interacts with three nucleotides of ssDNA or three base pairs of dsDNA. In the ssDNA complex structure the ATP-binding site is located at the subunit-subunit interface. The EcRecA-ssDNA nucleoprotein filament represents the structural intermediate responsible for homology pairing to a donor dsDNA, and the RecA-dsDNA structure is an end product after the strand exchange reaction [11].

The bacterial RecA monomer has three major domains: a small N-Terminal (NTD), a core ATPase domain and a large C-terminal domain (CTD). Members of RecA family, including the RadA protein of Archaea and the Rad51 and Dmc1 proteins of eukaryotes, share a core ATPase domain that contains the nucleotide binding site, the conserved Walker A and B motifs, and the putative DNA binding site(s), designed loops L_1_ and L_2_ [12–18].

The NTD of RecA family also shares an important domain responsible for self-polymerization of monomers in the presence or absence of DNA. The NTDs of the human Rad51 and the archaeal RadA protein have been both implicated in dsDNA binding [12,19], while RecA protein has an extra CTD for dsDNA binding [20]. The NTD of EcRecA protein structure contains 33 amino acids and assumes a conformation including an α-helix motif and a coil region. The coil region has a short β-loop polymerization motif, which interacts with a β-strand from the core ATPase domain, and is responsible for polymerization of the helical nucleoprotein filament in the compressed and extended RecA nucleoprotein conformations. Similar to the β-loop of RecA protein, RadA/Rad51/Dmc1 proteins have a β-polymerization motif in the NTD which together with a subunit rotation motif is responsible for structural transitions from an inactive hexameric ring to right and left—handed filaments [12,13,21,22].

The RecA protein can bind both ssDNA and dsDNA, but the nucleation occurs much more rapidly on ssDNA [8]. RecA protein nucleates onto DNA as oligomers of 4–6 subunits, and then the polymerization rapidly extends primarily in the 5′ to 3′ DNA direction at rates greater than 1000 monomers/min at 37°C [23–25]. Two conformations of helical RecA filaments have been structurally characterized. The first is an inactive and compressed form, formed by RecA alone or bound to ADP, either in the absence of DNA or bound to ssDNA or dsDNA. The second one is an active and extended filament, formed in the presence of DNA and ATP-γ-S or ATP [24,26–28].

In the present study, we report a functional and structural characterization of a RecA protein from *Herbaspirillum seropedicae* SmR1 (HsRecA), an endophytic bacterium capable of fixing nitrogen under ammonium and oxygen limiting conditions. Analysis of the *H*. *seropedicae* SmR1 genome sequence indicates the absence of *recF*, *dinI*, *dinB*, *recBCD*, *sbcA and sbcCD* genes and the presence of a truncated *umuC* gene [29,30], suggesting that RecA plays an important and novel role in DNA recombination and repair systems in this bacteria. Using ATPase, strand exchange assays and electron microscopy images, we compared wild type HsRecA and EcRecA protein activities, focusing on HsRecA protein binding onto ssDNA. We also determined the three-dimensional structure of HsRecA protein and explored the molecular mechanisms of polymerization of bacterial RecA as a helical nucleoprotein filament.

## Material and Methods

### Reagents

Restriction enzymes were purchased from New England Biolabs. Glycerol, Tris buffer were purchased from Fisher. All other reagents were obtained from Sigma unless otherwise described.

### Plasmid Construction, Cloning and Overexpression Host

The fragment *NdeI/BamHI* containing the wild type *recA* gene of *H*. *seropedicae* from the plasmid pAETWT-HMK [29] was cloned into *NdeI-BamHI* digested vector pET21a (Novagen). The integrity of the cloned fragment was confirmed by sequencing. *E*. *coli* STL2669, a nuclease-deficient strain, was used as host to overexpress the *H*. *seropedicae* RecA protein after 0.42 mM isopropyl-β-D-thiogalactopyranoside (IPTG) addition and 3–4 h incubation.

### Protein Purification

The native wild type EcRecA protein was purified using previously described protocols [31,32] and the native wild type HsRecA was purified as follows. All purification steps were carried out at 4°C. Cell paste (approximately 13 g) containing RecA protein was flash-frozen with liquid N_2_, then thawed overnight on ice in a lysis solution of 250 mM Tris-HCl (80% cation, pH 7.8) and 25% (w/v) sucrose, adjusting cell to 20% (w/v) ratio. The cells were frozen 2 times in N_2_, following addition of lysozyme solution (2.5 mg/mL final concentration lysozyme in 250 mM Tris-HCl (80% cation, pH 7.8), and addition of 0.02 mL of 500 mM EDTA per mL of final lysis solution. The lysate was sonicated for 20 min, using 30s on / 30s off cycle, 60% output, and then centrifuged per 1h30min to remove cells debris. DNA-binding proteins and HsRecA protein was precipitated from the lysate supernatant with addition of 0.111 mL of 5% (w/v) polyethyleneimine per mL of lysate and incubated for 1 h. The pellet was washed with R-buffer (20 mM Tris-HCl (80% cation, pH 7.8), 10% glycerol, 0.1 mM EDTA and 1 mM dithiothreitol) and then HsRecA protein was extracted from the pellet by addition of R-buffer + 300 mM ammonium sulfate two times.

The BioRex-70 (BioRad) (pH adjusted to 7.5 using 12 N HCl) was equilibrated with R-buffer + 300 mM ammonium sulfate and used to remove the excess of polyethyleneimine. The extracted HsRecA protein was then precipitated with 0.28 g/mL ammonium sulfate and centrifuged. The resulting pellet was washed twice with R-buffer + 0.28 g/mL ammonium sulfate. The pellet was resuspended in R—buffer + 50 mM KCl and loaded onto DEAE Sepharose fast flow column (GE Healthcare). HsRecA protein was eluted from the DEAE Sepharose column with a 7 column volume of a linear KCl gradient (0.05–1 M) in R-buffer. The eluted HsRecA protein was dialyzed into P-buffer (20 mM potassium phosphate, 10% glycerol, 0.1 mM EDTA and 1 mM dithiothreitol) and applied to ceramic hydroxyapatite column HAP (BIO-RAD). The HsRecA protein was eluted with a 10 column volumes of P-buffer containing a linear gradient of potassium phosphate (0.02–1 M). The most pure fractions containing HsRecA protein were pooled and dialyzed into R-buffer + 50 mM KCl and, then applied to Source 15-S column (GE Healthcare). HsRecA protein was not retained in the column and it was collected in the flow-through fraction. The pooled collected was applied directly into the Source 15-Q column (GE Healthcare) and submitted to a 10 column volume of a linear KCl gradient (0.05–1 M) in R-buffer. The fractions containing HsRecA protein were pooled, adjusted to 1 M ammonium sulfate and then applied to Butyl Sepharose column (GE Healthcare) which was submitted to a 10 column volume of a linear ammonium sulfate gradient (1–0 M) in R-buffer. The purified protein was free of detectable nuclease activity and showed 95% purity in SDS-PAGE. HsRecA and EcRecA concentration were determined using absorbance at 280 nm and the extinction coefficient 1.60×10^4^ and 2.23×10^4^ M^−1^cm^−1^, respectively.

*E*. *coli* single-stranded DNA binding protein (SSB) was purified as described previously [33] and its concentration was determined using an extinction coefficient of 2.83× 10^4^ M^−1^cm^−1^ at 280 nm.

### ATPase Assay

ATP hydrolysis was measured by a coupled spectrophotometric assay as previously described [34,35] at 37°C. The assays were carried out in a Varian Cary 300 dual beam spectrophotometer equipped with a temperature controller and a 12-position cell changer. Regeneration system of ATP (10 U of pyruvate kinase mL^−1^ and 3 mM phosphoenolpyruvate) from ADP and phosphoenolpyruvate is coupled to the conversion of NADH to NAD^+^ (2 mM NADH and 10 U of lactate dehydrogenase mL^−1^), which can be monitored by a decrease in absorbance at 380 nm. Although the maximum absorbance for NADH occurs at 340 nm, absorbances were measured at 380 nm to remain within the linear absorbance range of the spectrophotometer for the extended length of time required in these experiments. The amount of ATP hydrolyzed was calculated using the extinction coefficient of NADH, 1.21 mM^-1^cm^-1^. The standard reaction condition was RecA-buffer (25 mM Tris- OAc (80% cation, pH 7.5), 1 mM dithiothreitol, 5% (w/v) glycerol, 3 mM potassium glutamate, and 10 mM magnesium acetate). Concentrations of HsRecA, EcRecA, DNA, SSB, and ATP are indicated in figure legends. *E*. *coli* SSB was used to stimulates the ATPase activity of both HsRecA and EcRecA proteins when due.

### DNA Three Strand Exchange Reactions

Three strand exchange reactions were carried out as described previously [36,37] in a RecA-buffer at 37°C. An ATP regeneration system (10 U of pyruvate kinase mL^−1^ and 2.5 mM phosphoenolpyruvate) was also included. The EcRecA or HsRecA protein were pre-incubated with circular ssDNA M13mp18 (7249 mer) for 20 min, SSB protein and ATP were then added, followed by another 10 min of incubation. The reaction was initiated by the addition of M13mp18 linear dsDNA and incubated for 60 min. To stop the reaction, 5 μL of a stop and load reaction buffer (15% Ficoll, 0.25% bromophenol blue, 0.25% xylene cyanol, 25 mM EDTA, and 10% SDS) was added into each 10 μL aliquot collected. Samples were subjected to electrophoresis in 0.8% agarose gels with TAE buffer. Concentrations of HsRecA, EcRecA, DNA, SSB, and ATP are indicated in figure legends.

### Electron Microscopy

A modified alcian method was used to visualize RecA filaments. Activated grids were prepared as described previously [20]. Samples for electron microscopy analysis were prepared as follows. All incubations were carried out at 37°C. EcRecA or HsRecA (6.7 μM) was preincubated with 20 μM M13mp18 circular ssDNA in a RecA-buffer containing ATP regeneration system (10 U mL^-1^ creatine phosphokinase- PK and 12 mM phosphocreatine- PC) for 20 min. Then, 3 mM ATP with or without 2 μM SSB protein were added, and the reaction was incubated for another 10 min. To stabilize the filaments, 3 mM ATPγS was added and further incubated for 3 min.

The reaction mixture was diluted to a final DNA concentration of 0.4 ng/μL with diluting buffer (200 mM ammonium acetate, 10 mM Hepes and 10% glycerol (pH adjusted to 7.5)) and adsorbed to an activated alcian grid for 3 min. The grid was then touched to a drop of the diluting buffer followed by floating on a drop of the same buffer for 1 min. The sample was then stained by touching to a drop of 5% uranyl acetate followed by floating on a fresh drop of the same solution for 30 s. Finally, the grid was washed by touching to a drop of double distilled water followed by immersion in two 10 mL beakers of double distilled water. After the sample was dried, it was rotary-shadowed with platinum. This protocol is designed for visualization of complete reaction mixtures, and no attempt was made to remove unreacted material. Although this approach should yield results that give a true insight into reaction components, it does lead to samples with a high background of unreacted proteins. Imaging and photography were carried out with a TECNAI G2 12 Twin Electron Microscope (FEI Co.) equipped with a GATAN 890 CCD camera. Digital images of the nucleoprotein filaments were taken at X 15000 Magnification. Ten filaments each from EcRecA and HsRecA were measured three times using Metamorph analysis software and the average length was calculated in nm. The 0.5 μm scale bar was used as a standard to calculate the number of pixels per μm.

### Crystallization, Data Collection and Structure Refinement

All crystallization experiments were performed using the hanging-drop vapor diffusion method in 24-well plates at 20°C. All conditions were tested with the apo HsRecA form and with the ligands ATPγS and ADP. The initial crystallization screening was carried out using a reservoir consisting of 1 mL of each composition from the Cryo Suit (QIAGEN), PEG Ion, Natrix, Crystal Screen 1 and 2 and Crystal Screen Lite (Hampton Research) crystallization kits. Each drop contained 1 μL reservoir solution and an equal volume of the HsRecA protein solution (4 mg/mL) plus ligands when used. Clusters of multiple needle crystals were observed after 4 days under condition 0.2 M CaCl_2_, 20% w/v Polyethylene glycol 3,350 (PEG 3,350), pH 6.8. Further optimization of the condition was performed, initially varying the CaCl_2_ and PEG concentrations, and then screening for additives by using Additive Screens (Hampton Research). The best single crystals were finally obtained with 0.25 M CaCl_2_ and 14% w/v PEG 3,350 with further addition of Polypropylene glycol P 400 (PPG 400) onto the drop to give a final concentration of 5–8% w/v. Crystals from 0.1 mM HsRecA, 10 mM MgCl_2,_ 2 mM ADP solution reached dimensions of 0.8 mm × 0.2 mm × 0.2 mm after 4 days.

Diffraction data were collected using a Bruker diffractometer equipped with a rotating-anode X-ray generator operated at 40 kV and 40 mA and a Smart-6000 detector. The crystals were flash-cooled in liquid nitrogen at 100 K using the reservoir solution supplemented with 25% ethylene glycol as cryoprotectant. The crystals diffracted to a maximum resolution of 1.7 Å. A total of 1056 frames of data were collected with an oscillation angle of 0.5°, an exposure time of 120 s per frame and a crystal-to-detector distance of 60 mm.

Diffraction data were processed with the proteum2 software (Bruker AXS (2010) PROTEUM2, Version 2010.5, Bruker AXS Inc., Madison, Wisconsin, USA). Initial phasing was obtained by molecular replacement with the program Phaser [38]. Two domains of *E*. *coli* RecA (PDB entry 1xmv, residues 3 to 282, NTD and central ATPase domain, and residues 283 to 328, C-terminal) were used as separate search models. The initial structure was built with Phenix Autobuild [39] and it was completed by repeating cycles of manual model building with COOT [40] and refinement with Phenix.refine [41]. Translation, libration, and screw (TLS) groups were determined with TLSMD [42] and were used during refinement with Phenix.refine.

## Results

### ATPase Assay and Strand Exchange Activity

To investigate how HsRecA dynamically interacts with ssDNA the DNA-dependent ATPase activity of HsRecA was measured in a coupled spectrophotometric assay using circular ssDNA M13mp18 (cssDNA), ATP, an ATP regeneration system in the presence or absence of *E*. *coli* single-stranded binding protein (SSB). All ATPase assays were performed alongside EcRecA wild type in order to compare the two proteins’ activities.

**Fig 1:**
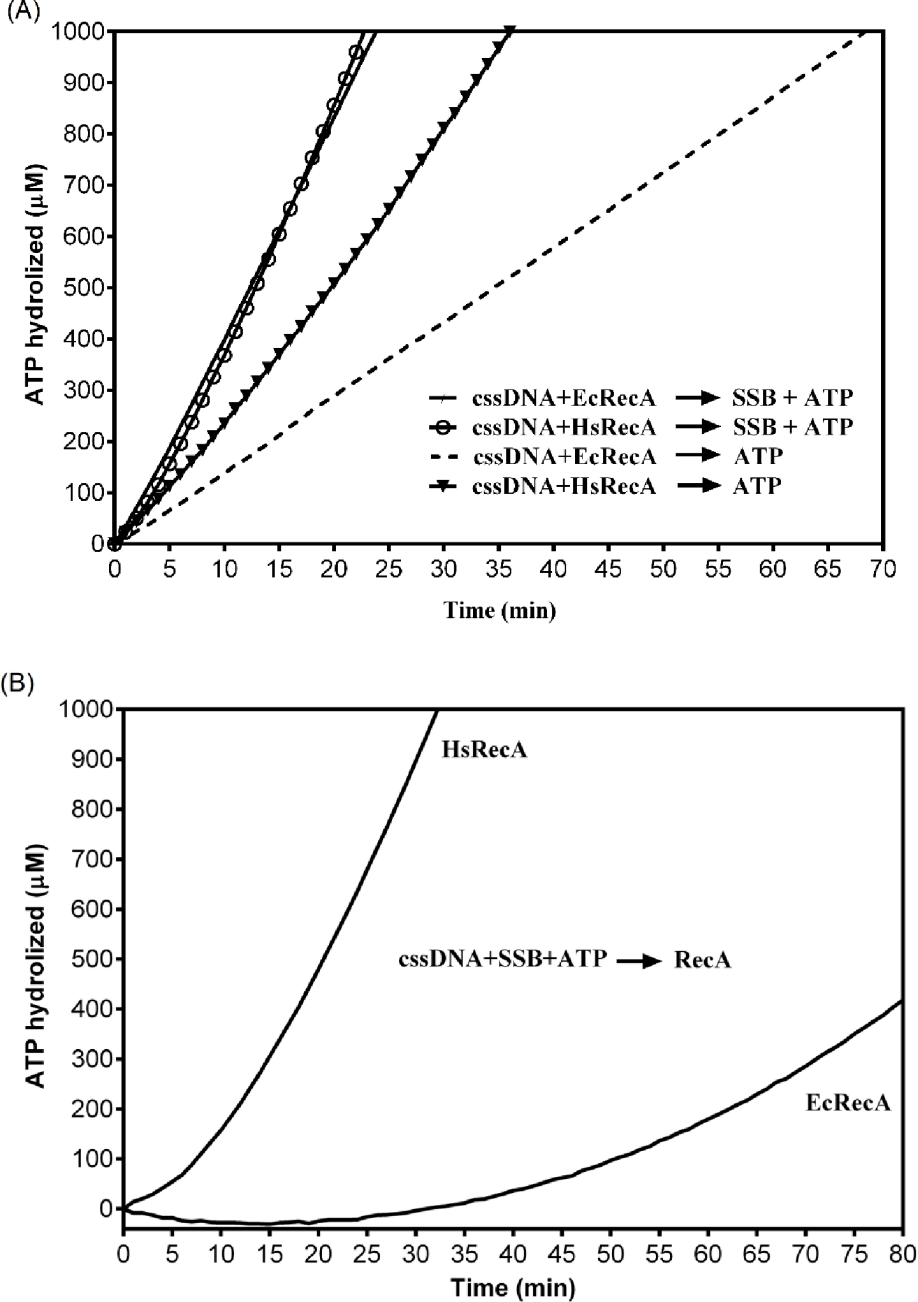
ATPase profile from EcRecA and HsRecA in the presence and absence of SSB protein. (A) Reaction 1: contained 5 μM nt M13mp18 cssDNA and 3 μM HsRecA or EcRecA, were previously incubated per 20 min at 37°C, following, 3 μM ATP and 0.5 μM SSB. Reaction 2: reaction 1 without SSB protein addition. Time 0 min indicates the addition of ATP and SSB. (B) The reaction contained 5 μM nt M13mp18 cssDNA, 3 μM ATP and 0.35 μM SSB, were previously incubated per 10 min at 37°C, following addition of 3 μM HsRecA or EcRecA (time 0 min).

A maximum steady state ATPase reaction is typically associated with the formation of complete and contiguous RecA filaments on the circular ssDNA [35]. The EcRecA and HsRecA apparent *k*_*cat*_ was determined after the steady-state rate of ATP hydrolysis was achieved, assuming one monomer of RecA bound for each three nucleotides of ssDNA. The results exhibited an apparent *k*_*cat*_ of 25.63 ± 0.10 and 28.91 ± 0.11 min^-1^, to EcRecA and HsRecA, respectively (Fig 1A–Reaction 1). The literature reports a *k*_*cat*_ of about 30 min^-1^ when EcRecA is bound to ssDNA under similar conditions [35,43,44]. In multiple trials, the ssDNA-dependent ATPase activity of the HsRecA protein was consistently about 10% higher than that exhibited by EcRecA protein.

The SSB protein is typically used in *in vitro* assays to remove the ssDNA secondary structures and to facilitate formation of contiguous RecA nucleoprotein filaments when it is added after DNA-RecA pre-incubation. However, when SSB is added prior to RecA, it strongly inhibits RecA filament nucleation and a long lag is seen before ATP hydrolysis is observed [45–47]. We utilized the EcSSB for all experiments, in part because the HsSSB was not available. Studies of RecA proteins from bacterial species ranging from *Neisseria gonorrhoeae* (Ng) to *Streptococcus pneumoniae* (Sp) to *Deinococcus radiodurans* (Dr) have failed to find any difference in SSB effects when EcSSB replaced cognate SSB proteins in standard RecA assays [48–50]. We note that the HsSSB protein is more closely related to the EcSSB (57% identity/69% similarity) than is the SSB from any of the three species mentioned above (50%/63%, 31%/50%, and 38%/49% for NgSSB, SpSSB, and DrSSB, respectively).

To evaluate the effects of SSB on the ATPase activity of HsRecA, we omitted SSB from the reaction described above (Fig 1A–Reaction 2). In this reaction HsRecA showed higher ATPase activity than EcRecA, with an apparent *k*_*cat*_ of 16.49 ± 0.11 and 8.89 ± 0.01 min^-1^, respectively. However, neither protein reached the same ATPase activity registered when SSB was present during the 70 minutes evaluated. In addition, a linear ATPase profile throughout the reaction indicated that there was no significant increase of RecA bound to the cssDNA over the course of the experiment.

We also investigated the influence of the moment that SSB is added in HsRecA activity. We added SSB, cssDNA and ATP 10 min before RecA addition, to allow SSB coating the cssDNA (Fig 1B). As already reported in the literature [45–47], EcRecA exhibited a low initial ATPase activity, followed by a very gradual increase, indicating a slow displacement of SSB. However, HsRecA protein displaced the SSB bound to the cssDNA much more effectively. The lag in HsRecA protein mediated ATP hydrolysis was short, and a high rate suggesting formation of contiguous HsRecA filaments was achieved within 10–15 min. HsRecA showed an apparent *k*_*cat*_ of 24.66 ± 1.45 min^-1^, almost as great as the *k*_*cat*_ determined in the previous assay when SSB was added after HsRecA-DNA pre-incubation. In comparison, EcRecA could not reach the same ATPase activity during the 80 min evaluated.

**Fig 2:**
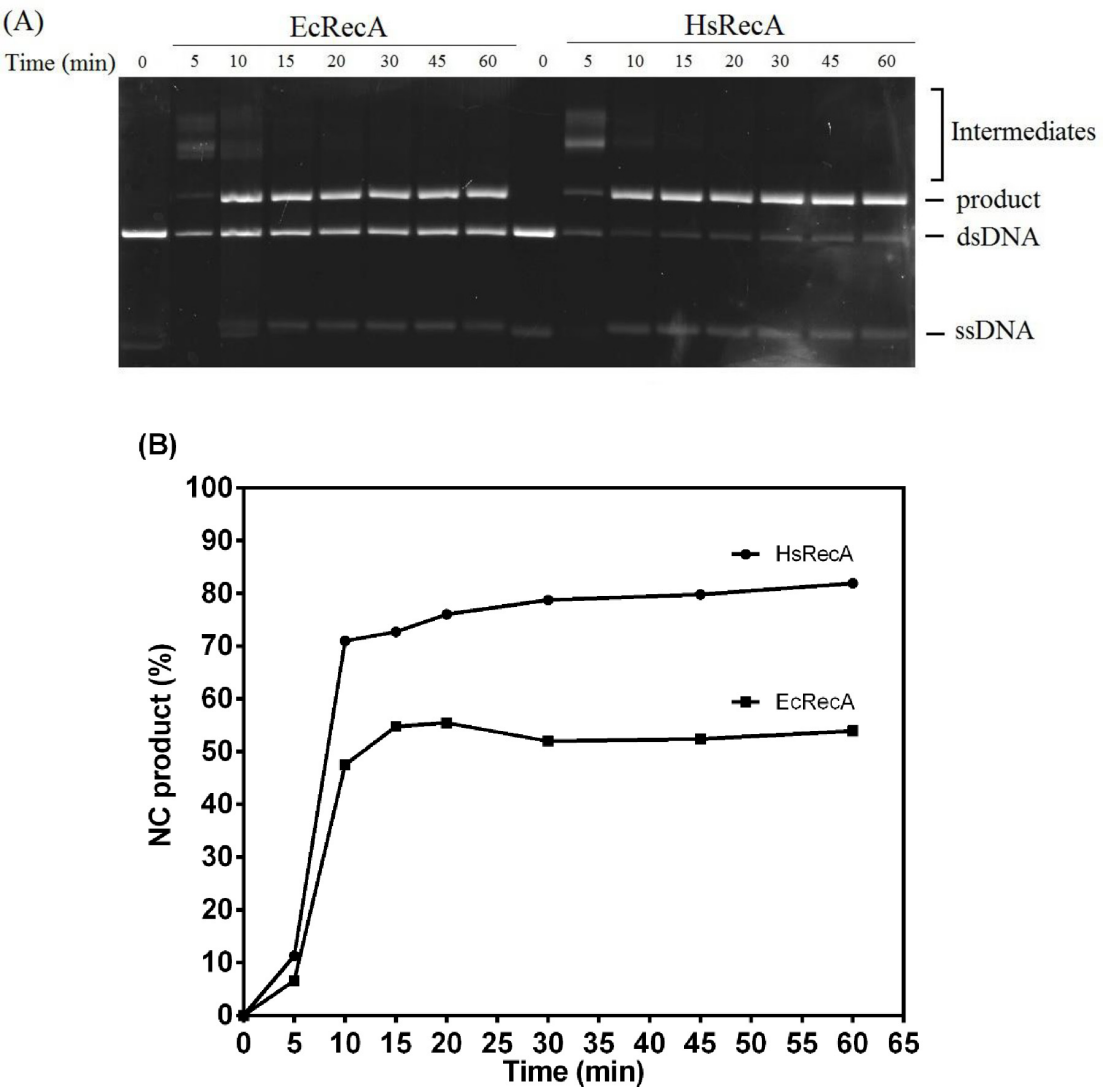
DNA strand exchange promoted by the wild-type HsRecA and EcRecA proteins. (A) The three DNA strand exchange containing 10 **μ**M nt M13mp18 cssDNA and 3.5 **μ**M HsRecA or EcRecA, were previously incubated per 20 min at 37°C, and then 3 **μ**M ATP and 1 **μ**M SSB were added and incubated for an additional 10 min. The minutes shown represents the time of reaction after addition of 20 **μ**M nt M13mp18 ldsDNA. (B) The percentage of duplex substrate converted into the nicked circular duplex (NC product) is plotted against the time.

We also examined the ability of the EcRecA and HsRecA proteins to catalyze strand exchange between cssDNA and linear dsDNA (ldsDNA) and form nicked circular dsDNA (NC product). Strand exchange promoted by HsRecA was significantly more efficient than the same reaction promoted by EcRecA. The reaction rates appeared to be similar for the reactions promoted by both proteins, with NC product detectable after 5 min incubation, as well as intermediates (Fig 2A). The reactions reached an apparent endpoint after 10 min. However, the extent of reaction was greater for the HsRecA protein at all time points (Fig 2B).

### Visualization of HsRecA Filaments by EM

**Fig 3:**
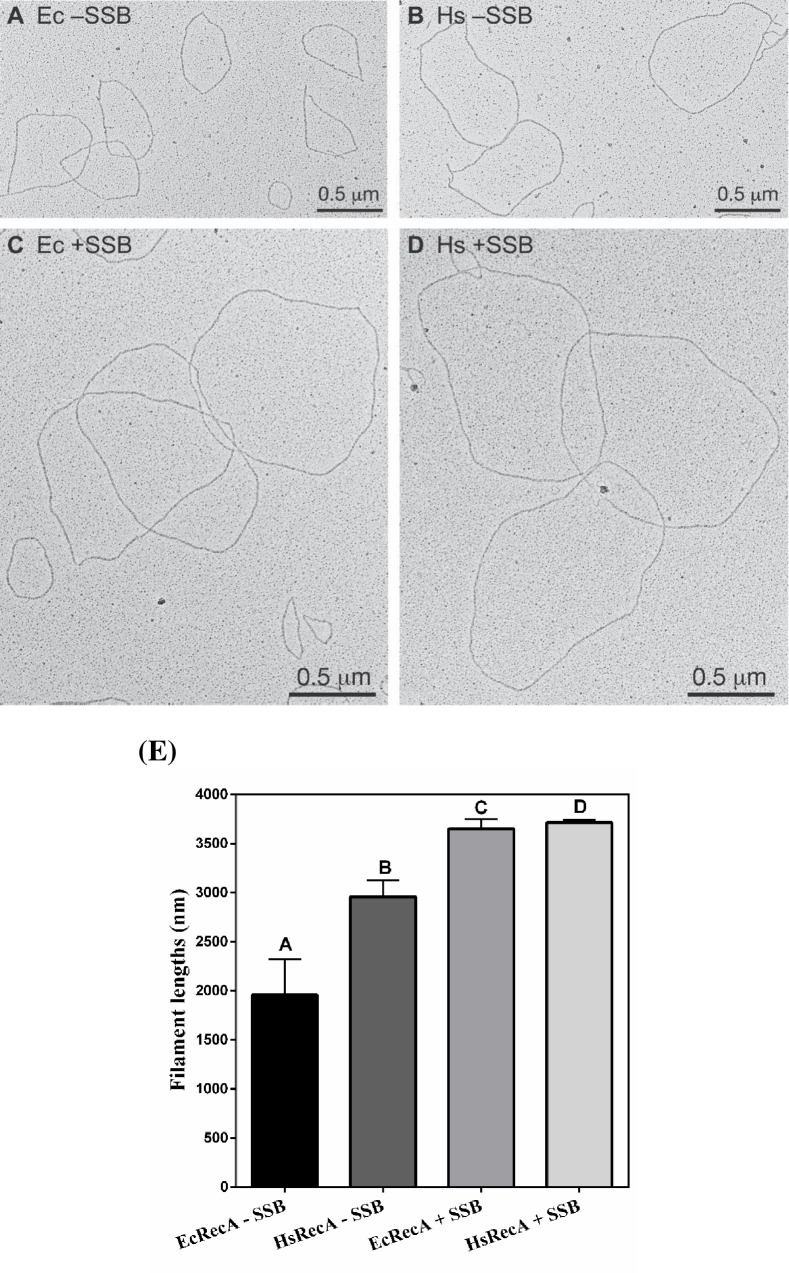
Electronic microscopy of EcRecA and HsRecA filaments in the absence or presence of EcSSB. (A) EcRecA-cssDNA filaments without EcSSB, (B) HsRecA-cssDNA filaments without EcSSB, (C) EcRecA-cssDNA filaments with EcSSB, (D) HsRecA-cssDNA filament with EcSSB, (E) Avarege length of 10 HsRecA and EcRecA filaments in the conditions assayed. Letters above each bar refer back to panels A-D. Reactions containing 6.7 **μ**M HsRecA or EcRecA and 20 **μ**M M13mp18 cssDNA were incubated at 37°C for 20 min, with or without 2 **μ**M Ec SSB. After 10 min, filaments were stabilized by a 3 min incubation with 3 **μ**M ATPγS and spread on an Alcian grid.

We used electron microscopy to evaluate the characteristics of HsRecA and EcRecA protein filaments in the absence of SSB (Fig 3A and 3B) and when it was added after RecA-cssDNA pre-incubation (Fig 3C and 3D).

In the absence of the EcSSB protein, EcRecA formed small filamented circles, with discontinuous regions being common. On the other hand, HsRecA protein formed more open filaments with fewer discontinuities. The addition of SSB eliminated detectable differences between the EcRecA and HsRecA filaments, forming large and completely filamented circles on the circular ssDNA substrate. Representative filaments are shown in Fig 3, panels A-D. The longer filaments formed by HsRecA in the absence of SSB indicate an enhanced capacity to extend filaments into regions of secondary structure. Note that in these images, only the RecA filaments are generally visible. The ssDNA is not seen unless it is bound with SSB (giving it a distinctive beaded appearance).

The general impressions gained from the initial survey of the grids was confirmed by direct filament length measurements. The length of ten filaments each from EcRecA and HsRecA-cssDNA filaments were measured in the presence and absence of SSB. In the presence of SSB, HsRecA and EcRecA exhibited filament lengths that were indistinguishable. There was no statistical significant difference (Tukey p<0.05) between HsRecA and EcRecA filaments lengths in the presence of SSB. On the other hand, in the absence of SSB, the HsRecA filaments were significantly longer than those formed by the EcRecA (Fig 3E).

### X-Ray Crystal Structure of HsRecA Protein

**Fig 4:**
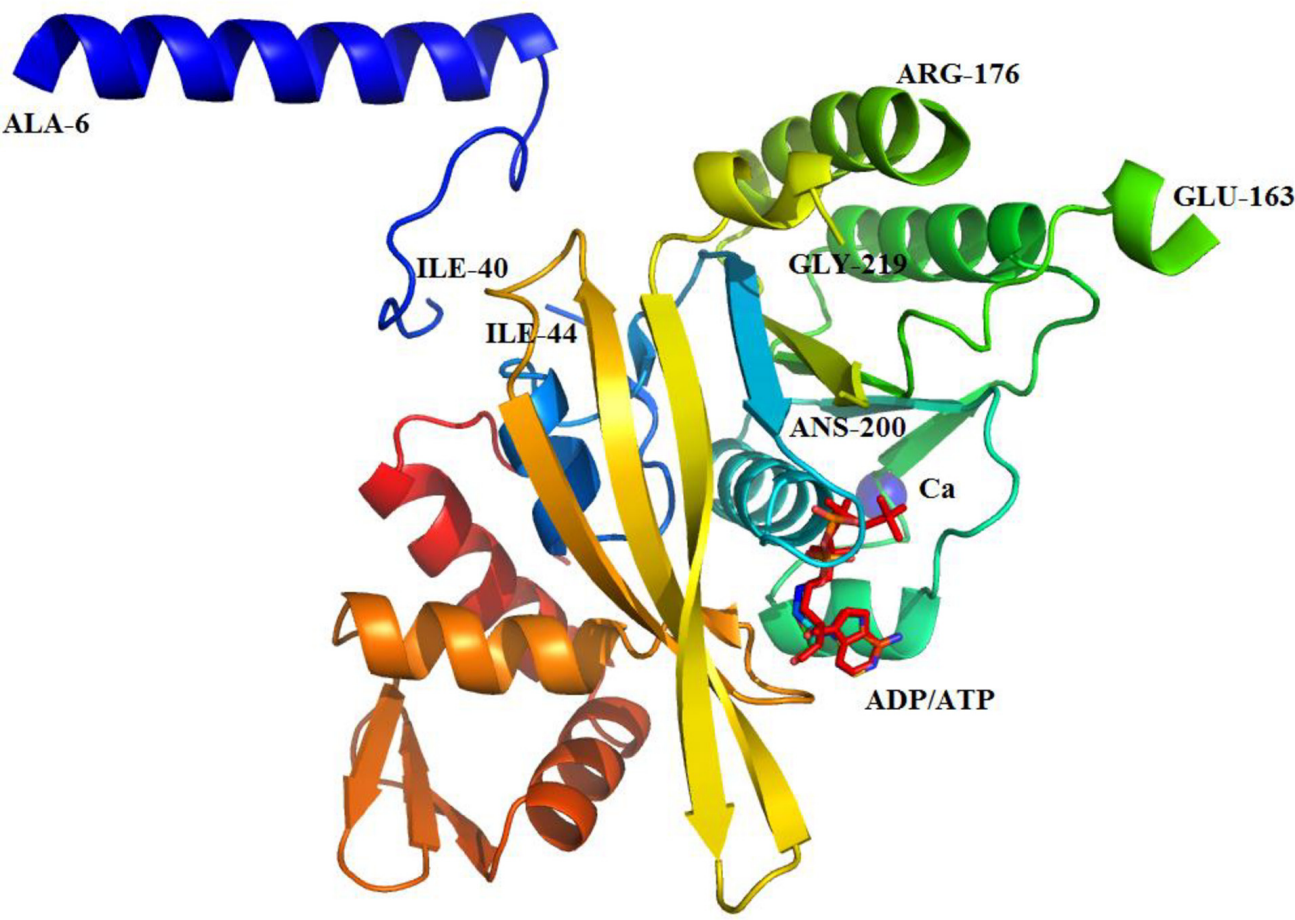
Ribbon diagram of the monomeric crystal structure of HsRecA protein. Regions that could be modeled were indicated by the last residue-number. HsRecA protein is composed of N-terminal domain (NTD), a central core ATPase domain and a large C-terminal domain (CTD). The core ATPase domain contains one Ca^2+^ ion (magenta sphere), coordinated by Asn119 and Asp120 and the ATPase activity site is partially occupied by ATP and ADP. Figure was prepared using the STRIDE program for secondary structure assignment [55], and visualized using PyMOL [56].

We solved the X-ray crystal structure of *H*. *seropedicae* RecA to 1.7 Å resolution (Fig 4). The structure was refined with good bond geometry and crystallographic quality statistics, with no residues in disallowed regions of the Ramachandran space (Table 1). The crystals contained a single HsRecA monomer per asymmetric unit.

**Table 1:** Data collection and refinement statistics.

	**HsRecA-ADP complex**
**Data collection**	
Wavelength (Å)	1.542
Resolution range (Å)*	75.71–1.70 (1.73–1.70)
Space group	P6_1_
Unit cell	
*a*, *b*, *c*, (Å)	87.41, 87.41, 91.35
α, β, γ (°)	90, 90, 120
Unique reflections	42574 (3467)
Multiplicity	5.18(1.83)
Completeness (%)	97.67 (79.50)
Mean I/σ(I)	31.03(2.08)
Wilson B-factor (Å^2^)	16.94
R-merge^†^	0.0467(0.6285)
**Refinement**	
Reflections used for R-free	2025(109)
R-work^‡^	0.1755 (0.3151)
R-free^‡^	0.2107 (0.3775)
Number atoms	
non-hydrogen	2743
macromolecules	2359
ligands	59
water	325
Protein residues	308
RMSD^§^	
Length bonds (Å)	0.015
Bond angles (°)	1.62
Ramachandran favored (%)	97.58%
Ramachandran allowed (%)	2.42%
Ramachandran outliers (%)	0
Clashscore	4.52
Average B-factor (Å^2^)	23.30
Macromolecules (Å^2^)	22.30
Ligands (Å^2^)	21.70
Solvent (Å^2^)	31.00

*Statistics for the highest-resolution shell are shown in parentheses.

^†^R_merge_ = Σ|I-<I>|/ΣI, where I is the observed intensity.

^‡^ R_work_ and R_free_ = Σ|F_obs_-F_calc_|/ΣF_obs_ where R_free_ was calculated over 5% of the amplitude chosen at random and not used in the refinement.

^§^RMSD, root-mean-square deviation.

Electron density maps derived from molecular replacement phasing permitted assembly of a model of the 351-residue HsRecA protein, with the exception of loops for which density was not observed (residues 1–5, 41–43, 164–175, 201–218, and 343–351). As seen in RecA protein structures from other bacterial species [10,11,27,51–53], the tertiary structure of HsRecA can be subdivided into three domains: a small N-terminal domain (NTD), a central core ATPase domain, and a large C-terminal domain (CTD) (Fig 4). The NTD (residues 6–40) consists of a α-helix and a random coil motif and the CTD (residues 276–343) consists of 3 α-helices and one antiparallel β-sheet motif. The core domain (residues 44–275) consists of a conserved α/β ATPase motif, which in its turn contains the Walker A (residues 72–81) and B (residues 147–157) motifs, and the disordered DNA binding loops L_1_ (residues 163–176) and L_2_ (residues 202–219) for which electron density was not observed in our structure. A Ca^2+^ ion is bound by Asn119 and Asp120 in the ATPase core. Proper Ca^2+^ coordination in the structure was confirmed by the CheckMyMetal web server [54].

The amino acid sequence alignment from HsRecA and EcRecA, shows that these two proteins shares 68.12% identity and 80.6% similarity. The overall structure of HsRecA is similar to that of EcRecA (PDB entry, 1XMV) with root-mean square deviation (RMSD) of 1.324 Å for Cα, 1.335 Å for backbone and 1.486 Å for all atoms. The structural comparison was performed using LSQKAB from the CCP4 program suite [57].

### ATPase Active Site

**Fig 5:**
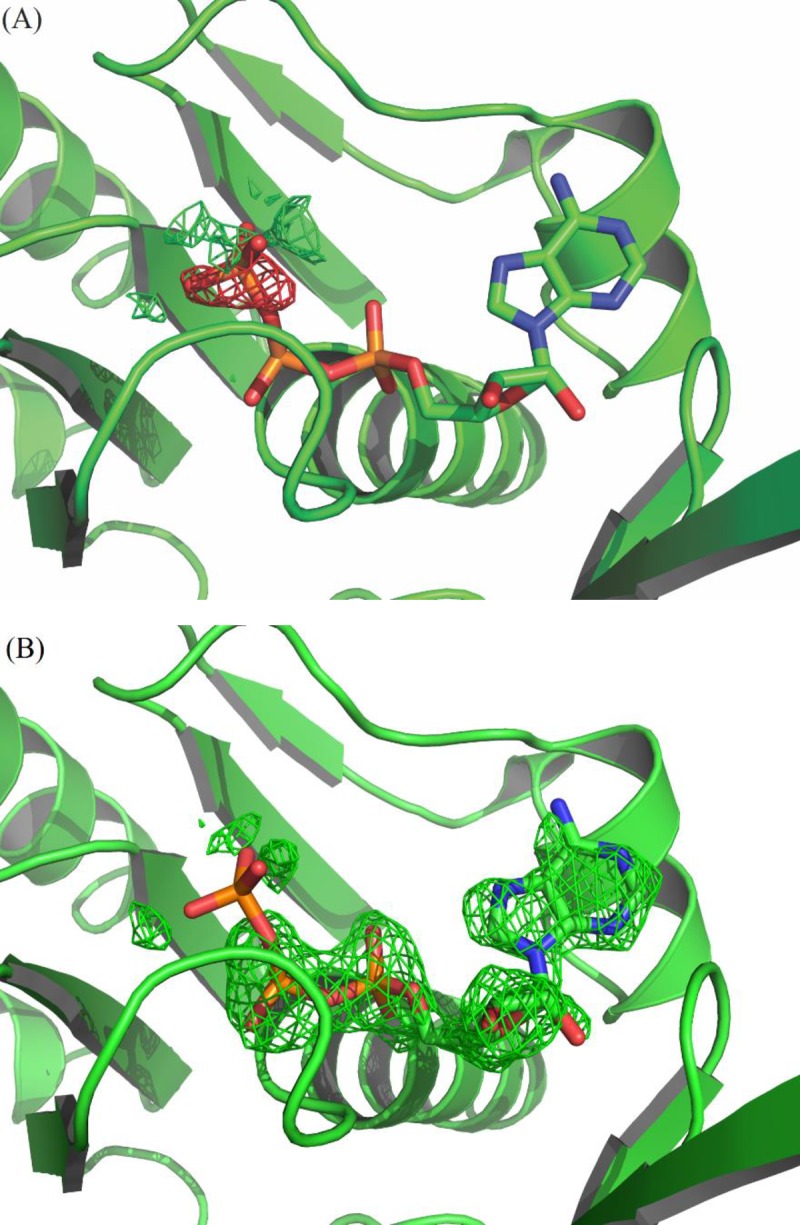
**Omit maps (mF**_**o**_**-DF**_**c**_**) at the ATP binding site:** (A) Refinement with ATP at 100% occupancy; the negative electron density over the γ-phosphate indicates that it should not be at this full occupancy. (B) Refinement with ATP at 39% occupancy; the positive electron density over the corresponding ADP moiety indicates that it should be at full occupancy. The omit maps are contoured at +3 (green) and -3 (red) σ levels.

The co-crystallized adenosine diphosphate (ADP) interacts directly with Ser76, Ser77, Thr80, Thr81, Asp107, Tyr110, and Gly272 in the HsRecA structure. Despite the fact that Mg^2+^ ion was present in the crystallization solution there was no electron density for this ion around the nucleotide ADP to suggest proper coordination. Mg^2+^ ion was also not observed in *Deinococcus radiodurans* and *Mycobacterium tuberculosis* RecA crystal structures [53,58]. We observed an extra electron density beyond the β-phosphate of ADP which could not be properly modeled with waters, SO_4_^3-^ or PO_4_^3-^ ions. The refinement of PO_4_^3-^ and ADP as the only nucleotide showed electron density that suggests them to be covalently linked, but there also remained residual negative electron density in the Fourier difference map over PO_4_^-3^ at 100% occupancy (Fig 5A and 5B). Therefore, we considered that this site is partially occupied by ATP, which might have come from the cells. The refinement with ADP and ATP sharing the position improved the adjustment to the electron density and resulted in occupancies of 61% and 39%, respectively. The amide nitrogen of Gln201 is at 2.88 Å from the γ-phosphate. This residue precedes the DNA binding Loop L_2_ and plays a role in the activation of the RecA function by induction of an allosteric effect of ATP [59]. Yet in the DrRecA-ATPγS complex this distance is 3.0 Å from the γ-thiophosphate. Both glutamines present the same conformation, different from the EcRecA structure (pdb entry 1xmv) which is bound to ADP.

### Assembly of HsRecA Protein in the Crystal Structure

**Fig 6:**
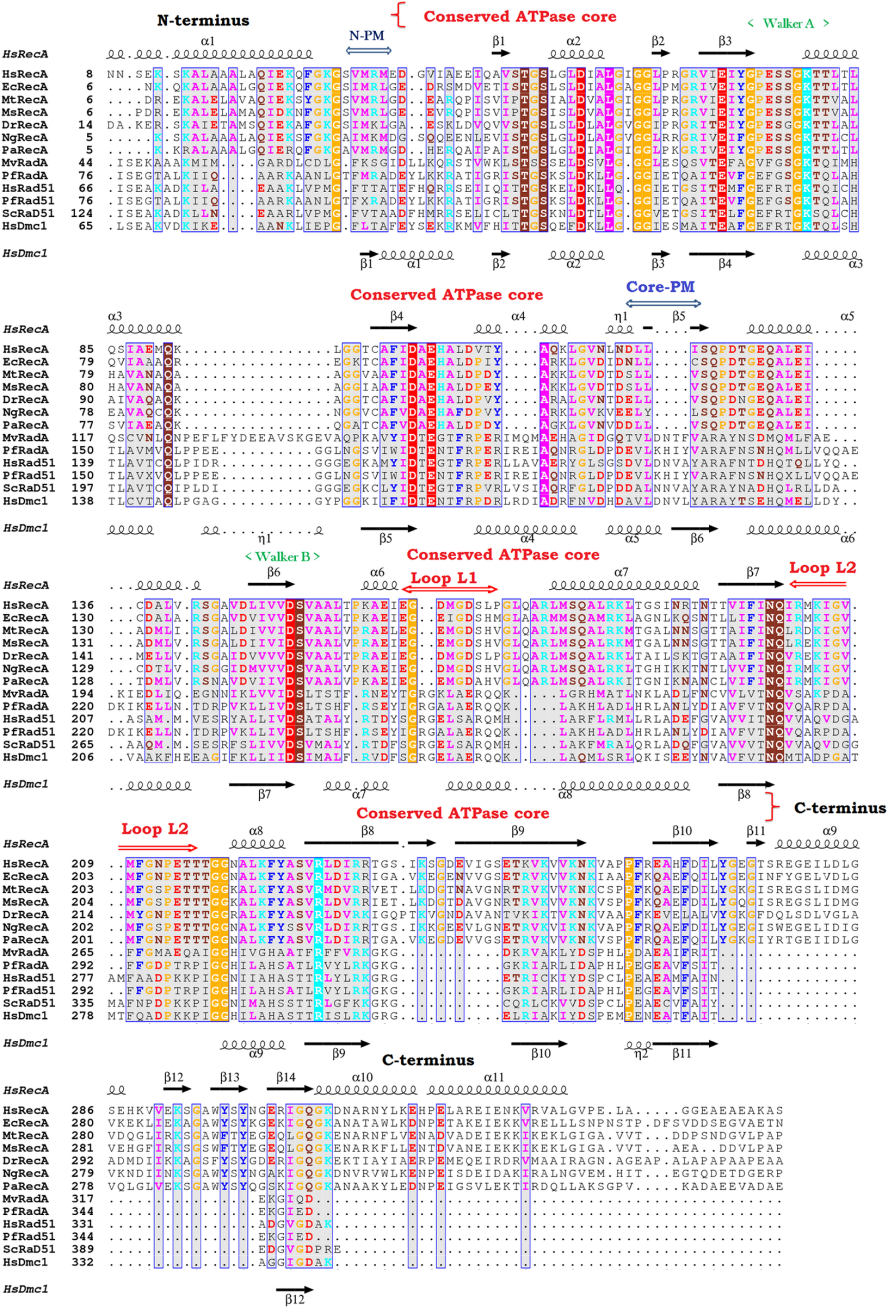
3D structural and amino acid sequence alignment from *H*. *seropedicae* (HsRecA), *E*. *coli* (EcRecA), *M*. *tuberculosis* (MtRecA), *M*. *smegmatis* (MsRecA), *D*. *radiodurans* (DrRecA), *N*. *gonorrhoeae* (NgRecA), *P*. *aeruginosa* (PaRecA), *M*. *voltae* (MvRadA), *P*. *furiosus* (RadA), *H*. *sapiens* (HsRad51), *P*. *furiosus* (Rad51), *S*. *cerevisiae* (ScRad51 and Dmc1). Part of the N-terminus has been removed for clarity. The position of the core ATPase domain and the C-terminus are indicated. Functional motifs are indicated above their corresponding amino acid sequences: N-PM and core-PM, N-terminal and core polymerization motif, respectively, the ATP binding Walker A and B motifs, the putative DNA binding sites Loop L_1_ and L_2_. The secondary structure of HsRecA and HsDmc1 are indicated at the top and bottom, respectively. The 3D structural and amino acid alignment was performed using the MultiProt and T-Coffee programs [61,62]. The results are visualized using ESPript—http://espript.ibcp.fr [63].

Bacterial RecA and other members of the RecA family (RadA, Dmc1 and Rad51) were aligned using their primary and tertiary structures to identify common regions (Fig 6). The monomer assembly regions were first identified in the RadA/Rad51/Dcm1 proteins [12,13,21,22,60]. They encompass residues in the NTD (Fig 6, N-PM region) and in the core ATPase domain (Fig 6, Core-PM region). The crystal structures of several RecA family members have revealed that the N-PM region is a polymerization motif, which is a short β-strand that just precedes the ATPase core and has conserved hydrophobic residues. Yet, using the alignment and the protein structures from the bacteria *E*. *coli* (pdb entry 3CMU, 1XMS, 1U98 and 1XMV), *D*. *radiodurans* (pdb entry 1XP8), *M*. *smegmatis* (pdb entry 1UBE, 1UBF and 1UBG), *M*. *tuberculosis* (pdb entry 1MO3, 1MO5, 1MO6 and 1G18) and ours, we noticed that this polymerization motif (N-PM) consists of a conserved sequence (Ser, Val/Ile, Met, Arg/Lys, Leu, Gly; residues 25–30 in the EcRecA sequence after elimination of the N-terminal Met, and residues 30–35 in the HsRecA sequence). This sequence interacts with the core ATPase domain (Core-PM) of the adjacent subunit at another conserved sequence (Asp, Asn, Leu, Leu, Val/Cys, Ser; residues 113–118 and 118–123 in the EcRecA and HsRecA sequences, respectively).

In the N-PM, the conserved hydrophobic residue phenylalanine found in RadA, Rad51 and Dmc1 proteins is replaced by a different hydrophobic residue, valine or isoleucine, in the RecA proteins. However, none of these substitutions affects a hydrogen bond with the Core-PM residues. The conserved methionine in the N-PM may play a role in binding/recognition of the hydrophobic β-strand polymerization motif in the core ATPase domain, promoting thus the polymerization of RecA filaments on the DNA substrate. The Table 2 shows the hydrogen bonds formed between the N-PM and the Core-PM from RecA proteins.

**Table 2:** Summary of hydrogen bonds formed between the N-PM and the Core-PM in RecA protein structures. The residues and their atoms involved in the interaction are indicated, as well as the atomic distances. Interactions were determined using the CCP4 application (Protein Interfaces, Surfaces and Assemblies—PISA) [52,64,65].

*H*. *seropedicae RecA*			*E*. *coli RecA*			*M*. *smegmatis RecA*			*M*. *tuberculosis RecA*			*D*. *radiodurans RecA*		
**N-PM residue [atom] d (Å) [atom] Core-PM residue**														
Ser31[O]	2.75	[Oγ]Ser124	Ser25[O]	2.63	[Oγ]Ser117	Ser37[O]	2.69	[Oγ]Ser129	Ser27[O]	2.49	[Oγ]Ser119	Ser26[O]	2.63	[Oγ]Ser118
									Ser27[O]	3.84	[N]Gln120			
Met33[N]	2.93	[O]Ile123	Met27[O]	2.65	[N]Cys116	Met39[N]	2.97	[O]Val128	Met29[N]	3.19	[O]Val118	Met28[O]	2.72	[N]Val117
Met33[O]	2.73	[N]Ile123	Met27[N]	2.65	[O]Cys116	Met39[O]	2.83	[N]Val128	Met29[O]	2.74	[N]Val118	Met28[N]	2.96	[O]Val117
Met35[N]	2.94	[O]Leu121				Lys40[Nζ]	2.75	[Oε2]Glu125				Arg29[Nη1]	3.89	[O]Ser114
			Leu29[N]	2.84	[O]Leu114	Leu41[N]	3.11	[O]Leu126	Leu31[N]	3.34	[O]Leu116	Leu30[N]	3.61	[O]Asp113
						Leu41[N]	3.83	[O]Asp124				Leu30[N]	3.33	[O]Leu115
												Leu30[N]	3.83	[O]Thr112
			Gly30[N]	2.99	[O]Asp112	Gly42[N]	3.35	[O]Asp124	Gly32[N]	3.35	[O]Asp114	Gly31[N]	2.85	[O]Asp113

**Fig 7:**
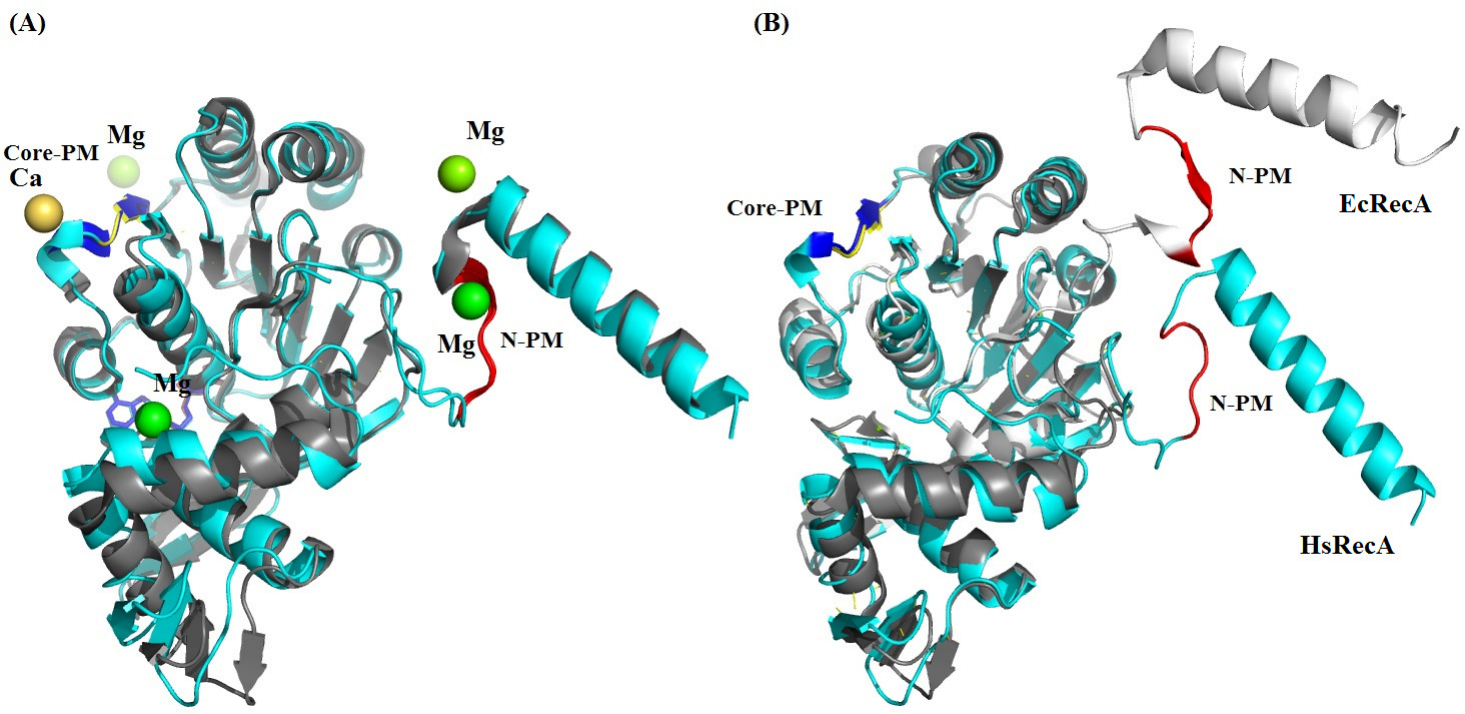
Superposition inactive and presynaptic monomers of EcRecA protein over the inactive HsRecA monomer. EcRecA protein is colored in gray and HsRecA in cyan. (A) Superposition of the inactive EcRecA structure (pdb entry 1XMV) over the HsRecA structure. Both structures have a β-loop motif in the N-PM. In the EcRecA structure, a Mg^2+^ ion interacts with the N-PM. In the HsRecA structure, a Ca^2+^ ion interacts with the Core-PM. (B) Superposition of presynaptic monomers of EcRecA structure (pdb entry 3CMU:A) over the HsRecA structure. The N-PM in the active EcRecA structure assumes a β-strand motif. The nucleotides ADP and ADP-AlF_4_, Mg^2+^ and Ca^2+^ ions, and the ssDNA bound in the EcRecA structure was removed for clarity. Figure was prepared using the STRIDE program for secondary structure assignment [55]. The superposition was generated using the MultiProt program [61] and visualized using PyMOL [56].

In all inactive RecA protein structures, the short β-strand motif becomes a β-loop motif conformation in the NTD (Fig 7A). This motif is observed in the HsRecA structure as well, although in the EcRecA-ssDNA presynaptic filament this stands as a β-strand motif, as found in the RecA family proteins (Fig 7B) and it is an antiparallel β-strand with respect to the second motif in the EcRecA-ssDNA pre-synaptic crystal structure filament.

The Ca^2+^ ion in the HsRecA Core-PM is coordinated by Asn119 and Asp120 and the Mg^2+^ ion in the EcRecA N-PM is coordinated by Glu18, Lys23 and Ile26 [27]. Both divalent ions may play a role in the formation and stability of RecA protein as a helical nucleoprotein filament. Previous studies showed that addition of excess Mg^2+^ (relative to the available ATP) helps to produce the active and extended conformation of the filament, required for DNA pairing and the strand exchange reaction *in vitro* [66]. HsRecA E36 is at the subunit interface border and might interact with excess ions along with other residues nearby, possibly increase the interaction within the subunit-subunit surface.

### Electrostatic Potential Surface

**Fig 8:**
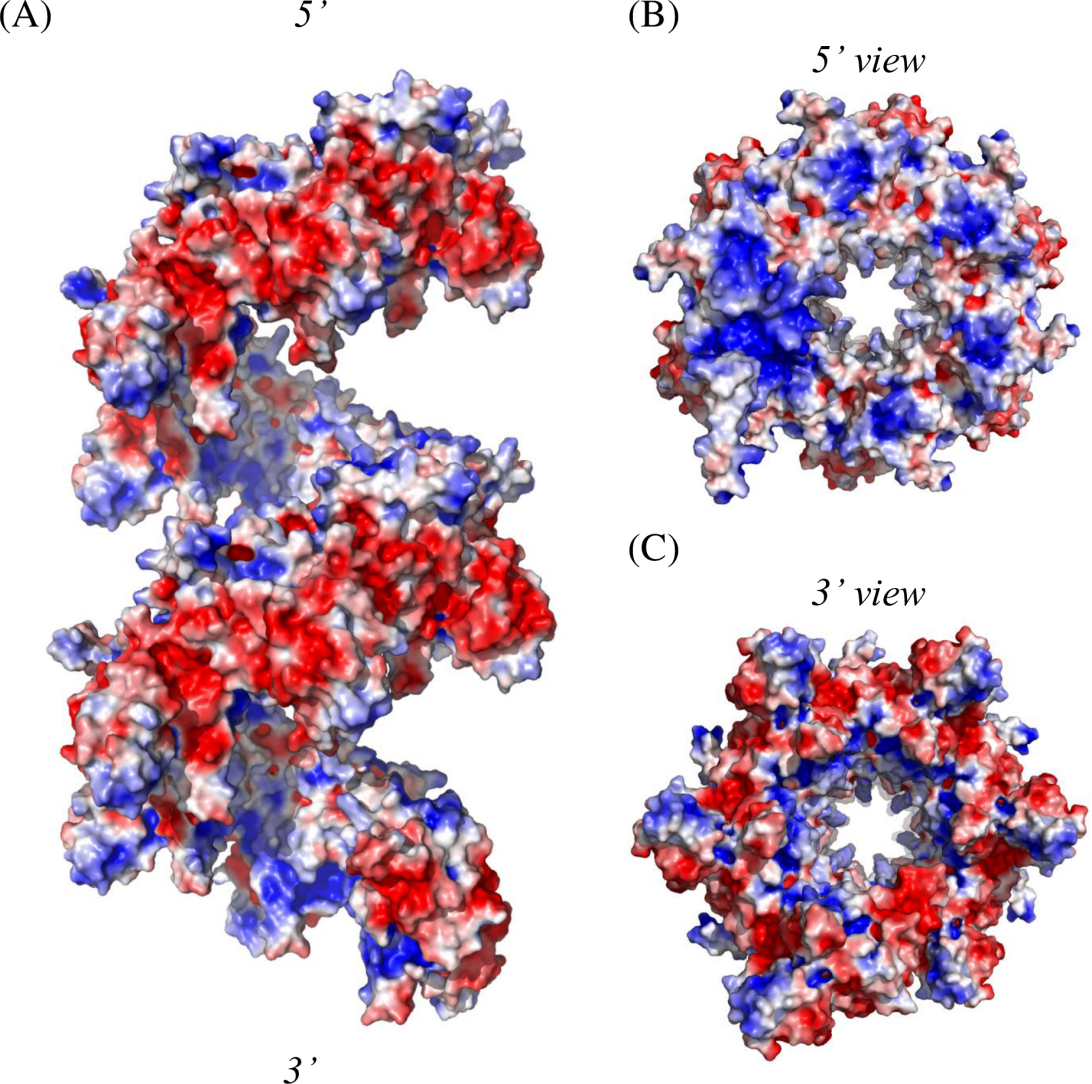
Electrostatic potential distribution on the solvent-accessible surface of HsRecA protein structure. (A) Side view of two subsequent helical hexamers. (B) and (C), 5′ and 3′ views, relative to the axial direction of the filament, respectively. The surface potential representation has charge levels from -**3*kT*/*e*** (red) to +**3*kT*/*e*** (blue). The electrostatic potential distribution was generated using the APBS program, side chain atom not ordered in the crystal were added using the PDB2PQR program and protonation states at pH 7.5 were assigned with the PROPKA program [67–69], and visualized using PyMOL [56].

We also reconstructed the HsRecA protein on the basis of its electrostatic potentials in order to understand the structural basis of their functional activities and subunit-subunit interactions. The electrostatic potential distribution on the solvent-accessible surface of the HsRecA protein for two subsequent helical hexamers is shown in Fig 8.

Due to the CTD portion of each monomer, there is a relatively large negatively charged region on the outer surface in the HsRecA filament structure. As has been observed for many RecA proteins, HsRecA protein has increased positive charge along the inner side of the filament, near the DNA binding sites (Fig 8B). There are subtle differences in the charge density and distribution relative to EcRecA protein,. These have not been analyzed in detail, but may eventually help explain the DNA binding properties of HsRecA protein. Important domains that affect filament polymerization stand orderly along the filament axis, such that there is a charge polarity change from negative to positive that binds the DNA from the 3′ to 5′ direction (Fig 8B and 8C). Therefore, we speculate that electrostatic interactions would facilitate proper filament assembly, after monomers had been stabilized by hydrophobic interactions between the N-PM of one monomer and the Core-PM of the subsequent one.

### Helical Pitch

**Fig 9:**
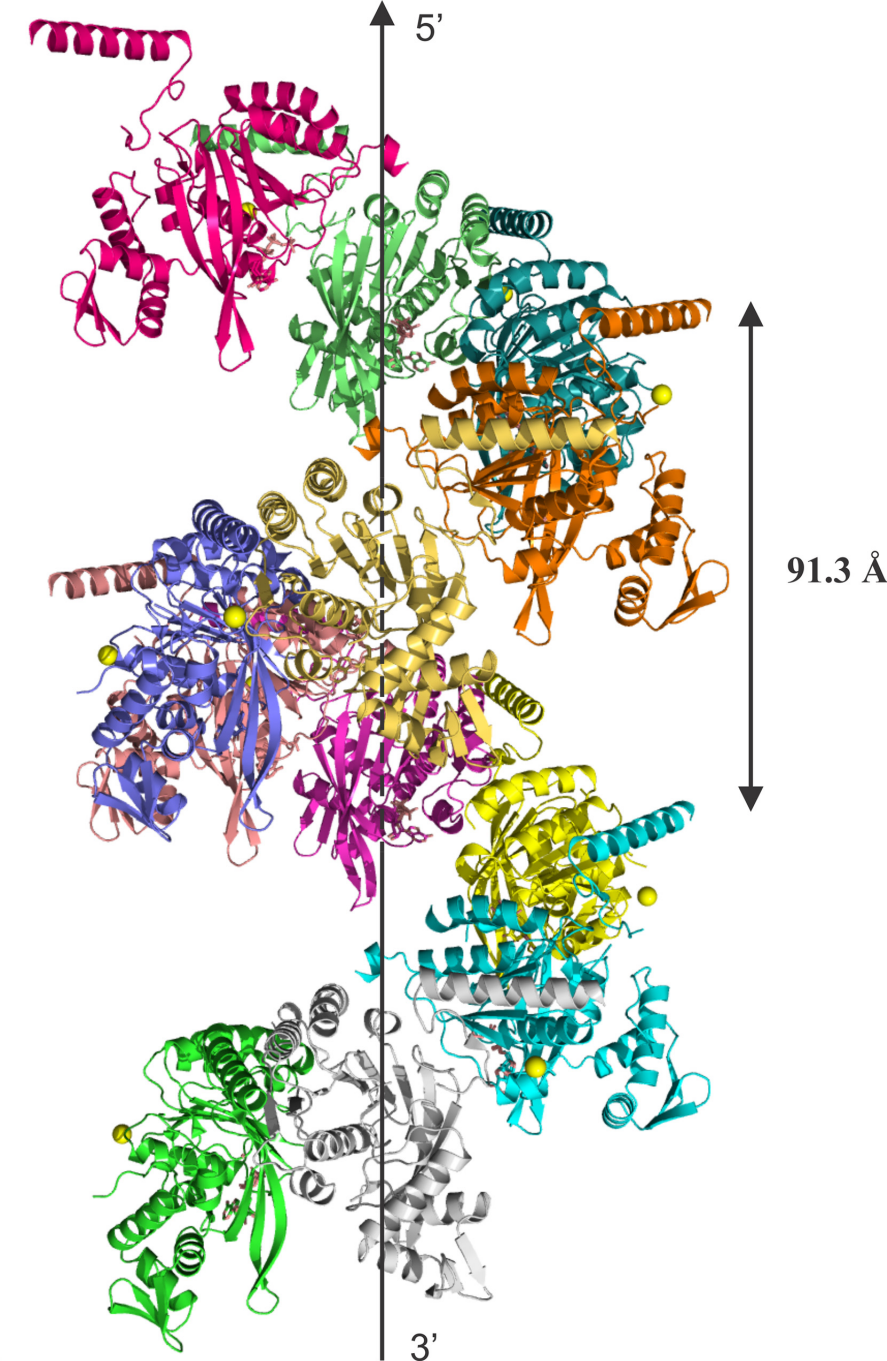
Ribbon diagram of two subsequent helical hexamers of HsRecA protein. Each monomer is colored differently. The hexameric structure has 6_1_-fold symmetry, the helical filament has a pitch of 91.3 Å. Figures were prepared using the STRIDE program for secondary structure assignment [55], and visualized using PyMOL [56].

The crystallography *6*_*1*_ symmetry leads to the formation of a helical filament that has a pitch of 91.3 Å (Fig 9), a value close to the pitch range 90–100 Å determined by electron microscopy for active filaments of EcRecA formed in the presence of DNA, ATP-γ-S or ATP [70]. Inactive and compressed filaments characterized to date have a helical pitch of 65–85 Å and are formed by RecA alone or bound to ADP, either in the absence of DNA or bound to ssDNA or dsDNA [10,26,51,53,58,71]. Despite the fact that HsRecA-ADP/ATP protein was crystallized with ADP and ATP, our structure presented a helical pitch characteristic of an active RecA filament form. However, as noted above, the structural packing is that of the inactive state.

## Discussion

In the present work, we report a functional and structural characterization of the RecA protein from *H*. *seropedicae*, an important bacterium to environmental crops in agricultural systems. The work has several conclusions. The HsRecA protein forms filaments on ssDNA, and promotes ATP hydrolysis and DNA strand exchange as do other bacterial RecA proteins. The HsRecA distinguishes itself with a somewhat more efficient DNA strand exchange reaction, and a moderately increased rate of ATP hydrolysis relative to that promoted by EcRecA protein. The HsRecA also displaces pre-bound SSB protein much faster than EcRecA. The structure of HsRecA protein, also presented here, is consistent with the RecA structures found for RecA proteins from other bacteria [10,11,27,51–53]. However, in the absence of DNA, the HsRecA crystal structure showed a helical pitch closer to the extended form of a RecA filament, although the structural packing remained that of the inactive state. Structural comparisons offer an opportunity to gain new structure-function insights.

The amino acid sequence alignment from *H*. *seropedicae* SSB and *E*. *coli* SSB, shows that these two proteins are 57.1% identical and 69.0% similar. Our results showed that the *E*. *coli* SSB protein stimulates the formation of contiguous HsRecA nucleoprotein filaments (Fig 1A–Reaction 1), as previously seen for *E*. *coli*, *Pseudomonas aeruginosa* and *Neisseria gonorrhoeae* RecA nucleoproteins [45,72,48,73]. However, the effect of SSB is different in *D*. *radiodurans*: either *E*. *coli* or *D*. *radiodurans* SSB protein suppresses DrRecA ATPase activity *in vitro* [74]. HsRecA reached an apparent *k*_*cat*_ of 28.91 ± 0.11 min^-1^. When compared under the same conditions, these rates were about 10% higher than those observed when EcRecA was bound to ssDNA.

The HsRecA protein has a higher capacity to bind to regions of secondary structure in ssDNA. Omission of the SSB from the ATPase assay decreases EcRecA ATPase activity about 65% (*k*_*cat*_ of 8.89 ± 0.01 min^-1^), while for HsRecA protein this reduction was about 43% (*k*_*cat*_ of 16.49 ± 0.11) (Fig 1A–Reaction 2). Based on the fact the ATPase activity is correlated to the amount of RecA bound to DNA, we suggest that HsRecA can better bind to the secondary structure of ssDNA in absence of SSB than the other proteins previously reported. Electron microscopy experiments performed to visualize EcRecA and HsRecA interaction with cssDNA and ATPγS also demonstrated a greater capacity to bind secondary structure for the HsRecA protein. When the SSB protein was absent, HsRecA filament lengths were significantly greater on M13mp18 ssDNA than those observed for EcRecA. When SSB protein was added after RecA and DNA pre-incubation, the filament lengths and characteristics were indistinguishable.

HsRecA protein displaces pre-bound SSB much more quickly than does EcRecA, and reaches a steady state with an apparent *k*_*cat*_ of 24.66 ± 1.45 min^-1^ within 10–15 min (Fig 1B). SSB protein is important in recombination processes because it stimulates the first phase, presynaptic polymerization of RecA protein on ssDNA, and the last phase, strand exchange [75–77]. Previous studies showed that some mutations in EcRecA (recA730 (E38K) [78], recA803 (V37M) [79], recA2020 (T121I) [80] and recA441 (E38K/I298V) [78,81,82]) can produce a similar capacity for bypassing the SSB block to nucleation. In HsRecA protein, these residues are conserved, except V37 and E38, which correspond to I44 and Q45 in HsRecA, respectively (Fig 6). Like EcRecA, the HsRecA has a C-terminus that is predominantly negatively charged (four Glu residues and one Lys among the 16 C-terminal amino acid residues. The concentration of negative charge is greater in the EcRecA C-terminus (7 of the last 17 amino acid residues, with no positive charges). Elimination of the EcRecA C-terminus also results in faster nucleation on SSB-coated ssDNA [20]. We have postulated that an interaction between the RecA C-terminus (unstructured in most RecA protein structures, including this one) and the surface at E38 could mask a RecA surface required for rapid SSB displacement. We postulate that the decrease in negative charge in the HsRecA, coupled with the amino acid residue substitutions at positions 44 and 45, may decrease the masking effect of the C-terminus and permit the observed rapid SSB displacement.

The monomeric structure of native HsRecA protein exhibits an architecture similar to that of bacterial RecA proteins previously crystallized. HsRecA protein has a small NTD, a core ATPase domain and a large CTD, with the same secondary structure elements of bacterial RecA proteins [9,51,53,58]. However, the crystallography *6*_*1*_ symmetry leads to the formation of a helical filament that has a pitch of 91.3 Å (Fig 9), a value within the pitch range of 90–100 Å determined by electron microscopy for active filaments of EcRecA formed in the presence of DNA, ATP-γ-S or ATP [70]. In contrast, the inactive and compressed filament has a helical pitch of 65–85 Å and is formed by RecA alone or bound to ADP in the absence of DNA [10,26,51,53,58,71]. Despite the fact that HsRecA-ADP/ATP protein formed an inactive filament in the presence of ADP and absence of DNA, our structure presented a helical pitch of an extended RecA filament form. Although previous studies using single particle analysis revealed that there is a considerable overlap in pitch between the active and inactive states [83], high salt concentrations induce RecA ATP hydrolysis and also facilitate the crystallization of the extended filament in the absence of ssDNA [84–86]. Curiously, polyethylene glycol (PEG) of various sizes also increased *M*. *voltae* RadA ATPase activity in the absence of DNA [87]. We suggest that the high concentrations of salt and PEG used in the crystallization solution of HsRecA (0.25 M CaCl_2,_ 14% w/v PEG 3,350 and 8% w/v PPG 400) could explain the crystallization of the HsRecA extended filament form even in the absence of DNA.

The mechanism of polymerization of RadA/Rad51 proteins as helical filaments have already been reported [17]. However, analyzing the primary and tertiary structures from bacterial RecA protein we found some differences. A conserved residue sequence in the NTD plays a role in the polymerization: hydrophobic residues from two motifs (N-PM and Core-PM) stabilize the subunit-subunit interaction in inactive structures. The N-PM assumes a β-loop conformation, while the Core-PM assumes a β-strand conformation. Sequence alignment revealed the presence of a conserved methionine in the bacterial N-PM RecAs, which we speculate may play a role in binding/recognition of the hydrophobic β-strand polymerization motif in the core ATPase domain. We also noticed that the transition of inactive and compressed to active and extended RecA structure follows the transition from a β-loop to a β-strand motif conformation in N-PM. The presence of the divalent ions Mg^2+^ and Ca^2+^ as observed in the N-PM and Core-PM, respectively, in EcRecA (pdb entry 1XMV) and HsRecA structures may play a role in the structural transition from compressed to extended conformation. This is similar to structural transitions in yeast Dmc1 protein, in which Ca^2+^ ion promotes the formation of an extended helical filament onto ssDNA [88].

The polymerization and dissociation of RecA protein from DNA are regulated by an array of proteins [6]. HsRecA was efficient at displacing SSB from ssDNA. In its absence, HsRecA was still able to bind more of the M13mp18 ssDNA circle, with its extensive secondary structure, than was observed with EcRecA when ATP or ATPγS is present. Most bacterial RecAs are dependent on RecO and RecR to displace SSB from DNA [89,90], but HsRecA protein appears to require less assistance. The *H*. *seropedicae* SSB, RecO and RecR proteins may play a somewhat different role in the recombination processes of this bacterium. Further experiments need to be performed to investigate the role of *H*. *seropedicae* SSB, RecO and RecR proteins in the mechanism of polymerization and stability of HsRecA filaments.

The HsRecA protein has a greater capacity to bind ssDNA in the presence of SSB, and this may reflect the plant-bacteria interaction and the apparent absence of some auxiliary proteins normally associated with RecA. RecA protein has recently been implicated in bacterial swarming, a flagellar-driven highly coordinated translocation of a bacterial colony across a moist surface [91,92]. This motility has been linked to a chemotaxis signaling pathway which includes methyl-accepting chemotaxis proteins (MCPs), CheW adaptor proteins and CheA kinase [93]. Irazoki *et al*. (2016) [94] showed that activation of the SOS response by the presence of a DNA-damaging compound increases the RecA concentration, thereby disturbing the equilibrium between RecA and CheW and resulting in the cessation of swarming. When the DNA-damaging source decreases or disappears, the repair of the DNA damage seems to restore colony swarming ability [94]. Based on the fact that *H*. *seropedicae* is subjected to many environmental factors that cause DNA-damage, such as solar ultraviolet radiation, reactive oxygen species, pH, among others, the enhanced capacity of HsRecA to bind DNA may facililtate more rapid repair and reestablishment of swarming, providing the bacteria a competitive advantage in colonization of plant root surfaces. Many genes related to chemotaxis were found in *H*. *seropedicae* SMR1 genome but further experiments are needed to evaluate the role of RecA to swarming in this bacterium.

## References

[1] Kowalczykowski SC, Eggleston a K. Homologous pairing and DNA strand-exchange proteins. Annu Rev Biochem. 1994;63: 991–1043. 10.1146/annurev.bi.63.070194.005015 7979259

[2] Cox MM. The bacterial RecA protein as a motor protein. Annu Rev Microbiol. 2003;57: 551–577. 10.1146/annurev.micro.57.030502.090953 14527291

[3] Janion C. Inducible SOS response system of DNA repair and mutagenesis in Escherichia coli. Int J Biol Sci. 2008;4: 338–44. 10.7150/ijbs.4.338 18825275 PMC2556049

[4] Cox MM. The Bacterial RecA Protein: Structure, Function, and Regulation Molecular Genetics of Recombination. Berlin, Heidelberg: Springer Berlin Heidelberg; 2007 pp. 53–94. 10.1007/4735_2006_0205

[5] Cox MM. Motoring along with the bacterial RecA protein. Nat Rev Mol Cell Biol. Nature Publishing Group; 2007;8: 127–138. 10.1038/nrm2099 17228330

[6] Cox MM. Regulation of Bacterial RecA Protein Function. Crit Rev Biochem Mol Biol. Taylor & Francis; 2008; 10.1080/10409230701260258 17364684

[7] Cox MM, Goodman MF, Kreuzer KN, Sherratt DJ, Sandler SJ, Marians KJ. The importance of repairing stalled replication forks. Nature. 2000;404: 37–41. 10.1038/35003501 10716434

[8] Lusetti SL, Cox MM. The bacterial RecA protein and the recombinational DNA repair of stalled replication forks. Annu Rev Biochem. 2002;71: 71–100. 10.1146/annurev.biochem.71.083101.133940 12045091

[9] Story RM, Weber IT, Steitz TA. The structure of the E. coli recA protein monomer and polymer. Nature. 1992;355: 318–325. 10.1038/355318a0 1731246

[10] Story RM, Steitz TA. Structure of the recA protein-ADP complex. Nature. 1992;355: 374–6. 10.1038/355374a0 1731253

[11] Chen Z, Yang H, Pavletich NP. Mechanism of homologous recombination from the RecA-ssDNA/dsDNA structures. Nature. 2008;453: 489–484. 10.1038/nature06971 18497818

[12] Chen LT, Ko TP, Chang YC, Lin KA, Chang CS, Wang AHJ, et al Crystal structure of the left-handed archaeal RadA helical filament: Identification of a functional motif for controlling quaternary structures and enzymatic functions of RecA family proteins. Nucleic Acids Res. 2007;35: 1787–1801. 10.1093/nar/gkl1131 17329376 PMC1874592

[13] Wu Y, He Y, Moya IA, Qian X, Luo Y. Crystal structure of archaeal recombinase RADA: a snapshot of its extended conformation. Mol Cell. 2004;15: 423–35. 10.1016/j.molcel.2004.07.014 15304222

[14] Kinebuchi T, Kagawa W, Enomoto R, Tanaka K, Miyagawa K, Shibata T, et al Structural Basis for Octameric Ring Formation and DNA Interaction of the Human Homologous-Pairing Protein Dmc1. Mol Cell. 2004;14: 363–374. 10.1016/S1097-2765(04)00218-7 15125839

[15] Okorokov AL, Chaban YL, Bugreev D V, Hodgkinson J, Mazin A V, Orlova E V. Structure of the hDmc1-ssDNA filament reveals the principles of its architecture. PLoS One. 2010;5: e8586 10.1371/journal.pone.0008586 20062530 PMC2797393

[16] Sandler SJ, Satin LH, Samra HS, Clark AJ. RecA-like genes from three archaean species with putative protein products similar to Rad51 and Dmc1 proteins of the yeast Saccharomyces cerevisiae. Nucleic Acids Res. 1996;24: 2125–2132. 10.1093/nar/24.11.2125 8668545 PMC145903

[17] Chang Y-W, Ko T-P, Lee C-D, Chang Y-C, Lin K-A, Chang C-S, et al Three new structures of left-handed RADA helical filaments: structural flexibility of N-terminal domain is critical for recombinase activity. PLoS One. 2009;4: e4890 10.1371/journal.pone.0004890 19295907 PMC2654063

[18] Wu Y, Qian X, He Y, Moya IA, Luo Y. Crystal structure of an ATPase-active form of Rad51 homolog from Methanococcus voltae. Insights into potassium dependence. J Biol Chem. 2005;280: 722–8. 10.1074/jbc.M411093200 15537659

[19] Aihara H, Ito Y, Kurumizaka H, Yokoyama S, Shibata T. The N-terminal domain of the human Rad51 protein binds DNA: structure and a DNA binding surface as revealed by NMR. J Mol Biol. 1999;290: 495–504. 10.1006/jmbi.1999.2904 10390347

[20] Eggler AL, Lusetti SL, Cox MM. The C terminus of the Escherichia coli RecA protein modulates the DNA binding competition with single-stranded DNA-binding protein. J Biol Chem. 2003;278: 16389–96. 10.1074/jbc.M212920200 12598538

[21] Lee C-D, Wang T-F. The N-terminal domain of Escherichia coli RecA have multiple functions in promoting homologous recombination. J Biomed Sci. 2009;16: 37 10.1186/1423-0127-16-37 19338667 PMC2672939

[22] Wang T-F, Chen L-T, Wang AH-J. Right or left turn? RecA family protein filaments promote homologous recombination through clockwise axial rotation. Bioessays. 2008;30: 48–56. 10.1002/bies.20694 18081011

[23] Bell JC, Plank JL, Dombrowski CC, Kowalczykowski SC. Direct imaging of RecA nucleation and growth on single molecules of SSB-coated ssDNA. Nature. Nature Publishing Group, a division of Macmillan Publishers Limited. All Rights Reserved.; 2012;491: 274–8. 10.1038/nature11598 23103864 PMC4112059

[24] Lindsleys E. Assembly and Disassembly of RecA Protein Filaments Opposite Filament Ends Occur at. 1990;265: 9043–9054. 2188972

[25] Shan Q, Bork JM, Webb BL, Inman RB, Cox MM. RecA protein filaments: end-dependent dissociation from ssDNA and stabilization by RecO and RecR proteins. J Mol Biol. 1997;265: 519–40. 10.1006/jmbi.1996.0748 9048946

[26] Galletto R, Amitani I, Baskin RJ, Kowalczykowski SC. Direct observation of individual RecA filaments assembling on single DNA molecules. Nature. 2006;443: 875–8. 10.1038/nature05197 16988658

[27] Xing X, Bell CE. Crystal structures of Escherichia coli RecA in a compressed helical filament. J Mol Biol. 2004;342: 1471–85. 10.1016/j.jmb.2004.07.091 15364575

[28] Roca AI, Cox MM. RecA protein: structure, function, and role in recombinational DNA repair. Prog Nucleic Acid Res Mol Biol. 1997;56: 129–223. Available: http://www.ncbi.nlm.nih.gov/pubmed/9187054. 9187054 10.1016/s0079-6603(08)61005-3

[29] Galvão CW, Souza EM, Etto RM, Pedrosa FO, Chubatsu LS, Yates MG, et al The RecX protein interacts with the RecA protein and modulates its activity in Herbaspirillum seropedicae. Braz J Med Biol Res. 2012;45: 1127–34. 10.1590/s0100-879x2012007500160 23044625 PMC3854219

[30] Pedrosa FO, Monteiro RA, Wassem R, Cruz LM, Ayub RA, Colauto NB, et al Genome of Herbaspirillum seropedicae strain SmR1, a specialized diazotrophic endophyte of tropical grasses. PLoS Genet. 2011;7: e1002064 10.1371/journal.pgen.1002064 21589895 PMC3093359

[31] Cox MM, McEntee K, Lehman IR. A simple and rapid procedure for the large scale purification of the recA protein of Escherichia coli. J Biol Chem. 1981;256: 4676–4678. Available: http://www.jbc.org/content/256/9/4676.abstract?ijkey=ebf61ddcdd61a793ee7acabcf72ece76a020aed5&keytype2=tf_ipsecsha. 7012155

[32] Shan Q, Cox MM, Inman RB. DNA Strand Exchange Promoted by RecA K72R: TWO REACTION PHASES WITH DIFFERENT Mg REQUIREMENTS. J Biol Chem. 1996;271: 5712–5724. 10.1074/jbc.271.10.5712 8621437

[33] Lohman TM, Green JM, Beyer RS. Large-scale overproduction and rapid purification of the Escherichia coli ssb gene product. Expression of the ssb gene under.lambda. PL control. Biochemistry. American Chemical Society; 1986;25: 21–25. 10.1021/bi00349a004 3006753

[34] Morrical SW, Lee J, Cox MM. Continuous association of Escherichia coli single-stranded DNA binding protein with stable complexes of recA protein and single-stranded DNA. Biochemistry. 1986;25: 1482–94. 10.1021/bi00355a003 2939874

[35] Brenner SL, Mitchell RS, Morrical SW, Neuendorf SK, Schutte BC, Cox MM. recA protein-promoted ATP hydrolysis occurs throughout recA nucleoprotein filaments. J Biol Chem. 1987;262: 4011–4016. 2951381

[36] Cox MM, Lehman IR. Directionality and polarity in recA protein-promoted branch migration. Proc Natl Acad Sci U S A. 1981;78: 6018–22. Available: http://www.pubmedcentral.nih.gov/articlerender.fcgi?artid=348968&tool=pmcentrez&rendertype=abstract. 6273839 10.1073/pnas.78.10.6018 PMC348968

[37] Bedale WA, Cox M. Evidence for the coupling of ATP hydrolysis to the final (extension) phase of RecA protein-mediated DNA strand exchange. J Biol Chem. 1996;271: 5725–32. 10.1074/jbc.271.10.5725 8621438

[38] McCoy AJ, Grosse-Kunstleve RW, Adams PD, Winn MD, Storoni LC, Read RJ. Phaser crystallographic software. J Appl Crystallogr. International Union of Crystallography; 2007;40: 658–674. 10.1107/S0021889807021206 19461840 PMC2483472

[39] Terwilliger TC, Grosse-Kunstleve RW, Afonine P V, Moriarty NW, Zwart PH, Hung LW, et al Iterative model building, structure refinement and density modification with the PHENIX AutoBuild wizard. Acta Crystallogr D Biol Crystallogr. International Union of Crystallography; 2008;64: 61–9. 10.1107/S090744490705024X 18094468 PMC2394820

[40] Emsley P, Lohkamp B, Scott WG, Cowtan K. Features and development of Coot. Acta Crystallogr D Biol Crystallogr. International Union of Crystallography; 2010;66: 486–501. 10.1107/S0907444910007493 20383002 PMC2852313

[41] Adams PD, Afonine P V, Bunkóczi G, Chen VB, Davis IW, Echols N, et al PHENIX: a comprehensive Python-based system for macromolecular structure solution. Acta Crystallogr D Biol Crystallogr. International Union of Crystallography; 2010;66: 213–21. 10.1107/S0907444909052925 20124702 PMC2815670

[42] Painter J, Merritt EA. Optimal description of a protein structure in terms of multiple groups undergoing TLS motion. Acta Crystallogr D Biol Crystallogr. International Union of Crystallography; 2006;62: 439–50. 10.1107/S0907444906005270 16552146

[43] Bianco PR, Weinstock GM. Interaction of the RecA protein of Escherichia coli with single-stranded oligodeoxyribonucleotides. Nucleic Acids Res. 1996;24: 4933–4939. 10.1093/nar/24.24.4933 9016663 PMC146329

[44] Weinstock G, McEntee K, Lehman I. Hydrolysis of nucleoside triphosphates catalyzed by the recA protein of Escherichia coli. Hydrolysis of UTP. J Biol Chem. 1981;256: 8856–8858. Available: http://www.jbc.org/content/256/16/8856.abstract?ijkey=fa6e333f30de803dbb1d35a529146ef725df2e16&keytype2=tf_ipsecsha. 7021554

[45] Kowalczykowski SC, Clow J, Somani R, Varghese A. Effects of the Escherichia coli SSB protein on the binding of Escherichia coli RecA protein to single-stranded DNA. Demonstration of competitive binding and the lack of a specific protein-protein interaction. J Mol Biol. 1987;193: 81–95. Available: http://www.ncbi.nlm.nih.gov/pubmed/3295259. 3295259 10.1016/0022-2836(87)90629-2

[46] Umezu K, Kolodner RD. Protein interactions in genetic recombination in Escherichia coli. Interactions involving RecO and RecR overcome the inhibition of RecA by single-stranded DNA-binding protein. J Biol Chem. 1994;269: 30005–13. Available: http://www.ncbi.nlm.nih.gov/pubmed/7962001. 7962001

[47] Shereda RD, Kozlov AG, Lohman TM, Cox MM, Keck JL. SSB as an organizer/mobilizer of genome maintenance complexes. Crit Rev Biochem Mol Biol. 2008;43: 289–318. 10.1080/10409230802341296 18937104 PMC2583361

[48] Stohl EA, Gruenig MC, Cox MM, Seifert HS. Purification and characterization of the RecA protein from Neisseria gonorrhoeae. PLoS One. Public Library of Science; 2011;6: e17101 10.1371/journal.pone.0017101 21359151 PMC3040777

[49] Steffen SE, Bryant FR. Purification and characterization of the RecA protein from Streptococcus pneumoniae. Arch Biochem Biophys. 2000;382: 303–9. 10.1006/abbi.2000.2029 11068882

[50] Kim J-I, Sharma AK, Abbott SN, Wood EA, Dwyer DW, Jambura A, et al RecA Protein from the extremely radioresistant bacterium Deinococcus radiodurans: expression, purification, and characterization. J Bacteriol. 2002;184: 1649–60. 10.1128/jb.184.6.1649-1660.2002 11872716 PMC134872

[51] Datta S, Krishna R, Ganesh N, Chandra NR, Muniyappa K, Vijayan M. Crystal structures of Mycobacterium smegmatis RecA and its nucleotide complexes. J Bacteriol. 2003;185: 4280–4. 10.1128/jb.185.14.4280-4284.2003 12837805 PMC164864

[52] Velankar S, Kleywegt GJ. The Protein Data Bank in Europe (PDBe): bringing structure to biology. Acta Crystallogr D Biol Crystallogr. 2011;67: 324–30. 10.1107/S090744491004117X 21460450 PMC3069747

[53] Rajan R, Bell CE. Crystal structure of RecA from Deinococcus radiodurans: insights into the structural basis of extreme radioresistance. J Mol Biol. 2004;344: 951–63. 10.1016/j.jmb.2004.09.087 15544805

[54] Zheng H, Chordia MD, Cooper DR, Chruszcz M, Müller P, Sheldrick GM, et al Validation of metal-binding sites in macromolecular structures with the CheckMyMetal web server. Nat Protoc. 2013;9: 156–170. 10.1038/nprot.2013.172 24356774 PMC4410975

[55] Frishman D, Argos P. Knowledge-based protein secondary structure assignment. Proteins. 1995;23: 566–79. 10.1002/prot.340230412 8749853

[56] Schrödinger L. The PyMOL Molecular Graphics System, Version 1.3r1. 2010 Aug.

[57] Winn MD, Ballard CC, Cowtan KD, Dodson EJ, Emsley P, Evans PR, et al Overview of the CCP4 suite and current developments. Acta Crystallogr D Biol Crystallogr. 2011;67: 235–42. 10.1107/S0907444910045749 21460441 PMC3069738

[58] Chandran A V, Prabu JR, Nautiyal A, Patil KN, Muniyappa K, Vijayan M. Structural studies on Mycobacterium tuberculosis RecA: molecular plasticity and interspecies variability. J Biosci. 2015;40: 13–30. 10.1007/s12038-014-9497-x 25740138

[59] Kelley JA, Knight KL. Allosteric Regulation of RecA Protein Function Is Mediated by Gln194. J Biol Chem. 1997;272: 25778–25782. 10.1074/jbc.272.41.25778 9325305

[60] Du L, Luo Y. Structure of a hexameric form of RadA recombinase from Methanococcus voltae. Acta Crystallogr Sect F Struct Biol Cryst Commun. International Union of Crystallography; 2012;68: 511–6. 10.1107/S1744309112010226 22691778 PMC3374503

[61] Shatsky M, Nussinov R, Wolfson HJ. A method for simultaneous alignment of multiple protein structures. Proteins. 2004;56: 143–56. 10.1002/prot.10628 15162494

[62] Notredame C, Higgins DG, Heringa J. T-Coffee: A novel method for fast and accurate multiple sequence alignment. J Mol Biol. 2000;302: 205–17. 10.1006/jmbi.2000.4042 10964570

[63] Robert X, Gouet P. Deciphering key features in protein structures with the new ENDscript server. Nucleic Acids Res. 2014;42: W320–4. 10.1093/nar/gku316 24753421 PMC4086106

[64] Krissinel E, Henrick K. Inference of macromolecular assemblies from crystalline state. J Mol Biol. 2007;372: 774–97. 10.1016/j.jmb.2007.05.022 17681537

[65] Krissinel E. Macromolecular complexes in crystals and solutions. Acta Crystallogr D Biol Crystallogr. 2011;67: 376–85. 10.1107/S0907444911007232 21460456 PMC3069753

[66] Lusetti SL, Shaw JJ, Cox MM. Magnesium ion-dependent activation of the RecA protein involves the C terminus. J Biol Chem. 2003;278: 16381–8. 10.1074/jbc.M212916200 12595538

[67] Dolinsky TJ, Czodrowski P, Li H, Nielsen JE, Jensen JH, Klebe G, et al PDB2PQR: expanding and upgrading automated preparation of biomolecular structures for molecular simulations. Nucleic Acids Res. 2007;35: W522–5. 10.1093/nar/gkm276 17488841 PMC1933214

[68] Dolinsky TJ, Nielsen JE, McCammon JA, Baker NA. PDB2PQR: an automated pipeline for the setup of Poisson-Boltzmann electrostatics calculations. Nucleic Acids Res. 2004;32: W665–7. 10.1093/nar/gkh381 15215472 PMC441519

[69] Baker NA, Sept D, Joseph S, Holst MJ, McCammon JA. Electrostatics of nanosystems: application to microtubules and the ribosome. Proc Natl Acad Sci U S A. 2001;98: 10037–41. 10.1073/pnas.181342398 11517324 PMC56910

[70] Egelman EH, Stasiak A. Structure of helical RecA-DNA complexes. Complexes formed in the presence of ATP-gamma-S or ATP. J Mol Biol. 1986;191: 677–97. Available: http://www.ncbi.nlm.nih.gov/pubmed/2949085. 2949085 10.1016/0022-2836(86)90453-5

[71] Ellouze C, Takahashi M, Wittung P, Mortensen K, Schnarr M, Norden B. Evidence for Elongation of the Helical Pitch of the RecA Filament Upon ATP and ADP Binding Using Small-Angle Neutron Scattering. Eur J Biochem. 1995;233: 579–583. 10.1111/j.1432-1033.1995.579_2.x 7588804

[72] Roy R, Kozlov AG, Lohman TM, Ha T. SSB protein diffusion on single-stranded DNA stimulates RecA filament formation. Nature. Macmillan Publishers Limited. All rights reserved; 2009;461: 1092–7. 10.1038/nature08442 19820696 PMC2782680

[73] Baitin DM, Bakhlanova I V, Kil Y V, Cox MM, Lanzov VA. Distinguishing characteristics of hyperrecombinogenic RecA protein from Pseudomonas aeruginosa acting in Escherichia coli. J Bacteriol. 2006;188: 5812–20. 10.1128/JB.00358-06 16885449 PMC1540092

[74] Ngo K V, Molzberger ET, Chitteni-Pattu S, Cox MM. Regulation of Deinococcus radiodurans RecA protein function via modulation of active and inactive nucleoprotein filament states. J Biol Chem. American Society for Biochemistry and Molecular Biology; 2013;288: 21351–66. 10.1074/jbc.M113.459230 23729671 PMC3774403

[75] Radding CM. Homologous pairing and strand exchange in genetic recombination. Annu Rev Genet. Annual Reviews 4139 El Camino Way, P.O. Box 10139, Palo Alto, CA 94303–0139, USA; 1982;16: 405–37. 10.1146/annurev.ge.16.120182.002201 6297377

[76] Radding CM, Flory J, Wu A, Kahn R, DasGupta C, Gonda D, et al Three phases in homologous pairing: polymerization of recA protein on single-stranded DNA, synapsis, and polar strand exchange. Cold Spring Harb Symp Quant Biol. 1983;47 Pt 2: 821–8. Available: http://www.ncbi.nlm.nih.gov/pubmed/6345077. 10.1101/sqb.1983.047.01.094 6345077

[77] Egner C, Azhderian E, Tsang SS, Radding CM, Chase JW. Effects of various single-stranded-DNA-binding proteins on reactions promoted by RecA protein. J Bacteriol. 1987;169: 3422–8. Available: http://www.pubmedcentral.nih.gov/articlerender.fcgi?artid=212412&tool=pmcentrez&rendertype=abstract. 3301800 10.1128/jb.169.8.3422-3428.1987 PMC212412

[78] Wang TC, Chang HY, Hung JL. Cosuppression of recF, recR and recO mutations by mutant recA alleles in Escherichia coli cells. Mutat Res. 1993;294: 157–66. 10.1016/0921-8777(93)90024-b 7687008

[79] Madiraju M V, Templin A, Clark AJ. Properties of a mutant recA-encoded protein reveal a possible role for Escherichia coli recF-encoded protein in genetic recombination. Proc Natl Acad Sci U S A. 1988;85: 6592–6. 10.1073/pnas.85.18.6592 2842780 PMC282023

[80] Wang TC, Smith KC. recA (Srf) suppression of recF deficiency in the postreplication repair of UV-irradiated Escherichia coli K-12. J Bacteriol. 1986;168: 940–6. Available: http://www.pubmedcentral.nih.gov/articlerender.fcgi?artid=213575&tool=pmcentrez&rendertype=abstract. 3023291 10.1128/jb.168.2.940-946.1986 PMC213575

[81] Thomas A, Lloyd RG. Control of recA dependent activities in Escherichia coli: a possible role for the recF product. J Gen Microbiol. 1983;129: 681–6. 10.1099/00221287-129-3-681 6348206

[82] Volkert MR, Margossian LJ, Clark AJ. Two-component suppression of recF143 by recA441 in Escherichia coli K-12. J Bacteriol. 1984;160: 702–5. Available: http://www.pubmedcentral.nih.gov/articlerender.fcgi?artid=214793&tool=pmcentrez&rendertype=abstract. 6094485 10.1128/jb.160.2.702-705.1984 PMC214793

[83] Yu X, Jacobs SA, West SC, Ogawa T, Egelman EH. Domain structure and dynamics in the helical filaments formed by RecA and Rad51 on DNA. Proc Natl Acad Sci U S A. 2001;98: 8419–24. 10.1073/pnas.111005398 11459984 PMC37452

[84] Hewat EA, Ruigrok RW, DiCapua E. Activation of recA protein: the pitch of the helical complex with single-stranded DNA. EMBO J. 1991;10: 2695–8. Available: http://www.pubmedcentral.nih.gov/articlerender.fcgi?artid=452972&tool=pmcentrez&rendertype=abstract. 1868839 10.1002/j.1460-2075.1991.tb07813.x PMC452972

[85] DiCapua E, Cuillel M, Hewat E, Schnarr M, Timmins PA, Ruigrok RW. Activation of recA protein. The open helix model for LexA cleavage. J Mol Biol. 1992;226: 707–19. Available: http://www.ncbi.nlm.nih.gov/pubmed/1507222. 1507222 10.1016/0022-2836(92)90627-v

[86] Pugh BF, Cox MM. General mechanism for RecA protein binding to duplex DNA. J Mol Biol. 1988;203: 479–93. 10.1016/0022-2836(88)90014-9 3058986

[87] Wu Y, He Y, Moya IA, Qian X, Luo Y. Crystal structure of archaeal recombinase RADA: a snapshot of its extended conformation. Mol Cell. 2004;15: 423–35. 10.1016/j.molcel.2004.07.014 15304222

[88] Lee M-H, Chang Y-C, Hong EL, Grubb J, Chang C-S, Bishop DK, et al Calcium ion promotes yeast Dmc1 activity via formation of long and fine helical filaments with single-stranded DNA. J Biol Chem. 2005;280: 40980–4. 10.1074/jbc.M505896200 16204247

[89] Bork JM. The RecOR proteins modulate RecA protein function at 5’ ends of single-stranded DNA. EMBO J. 2001;20: 7313–7322. 10.1093/emboj/20.24.7313 11743007 PMC125792

[90] Inoue J, Nagae T, Mishima M, Ito Y, Shibata T, Mikawa T. A mechanism for single-stranded DNA-binding protein (SSB) displacement from single-stranded DNA upon SSB-RecO interaction. J Biol Chem. 2011;286: 6720–32. 10.1074/jbc.M110.164210 21169364 PMC3057862

[91] Mayola A, Irazoki O, Martínez IA, Petrov D, Menolascina F, Stocker R, et al RecA Protein Plays a Role in the Chemotactic Response and Chemoreceptor Clustering of Salmonella enterica. Cloeckaert A, editor. PLoS One. Public Library of Science; 2014;9: e105578 10.1371/journal.pone.0105578 25147953 PMC4141790

[92] Gómez-Gómez J-M, Manfredi C, Alonso J-C, Blázquez J, Harshey R, Soutourina O, et al A novel role for RecA under non-stress: promotion of swarming motility in Escherichia coli K-12. BMC Biol. BioMed Central; 2007;5: 14 10.1186/1741-7007-5-14 17391508 PMC1852089

[93] Burkart M, Toguchi A, Harshey RM. The chemotaxis system, but not chemotaxis, is essential for swarming motility in Escherichia coli. Proc Natl Acad Sci U S A. 1998;95: 2568–73. Available: http://www.ncbi.nlm.nih.gov/pubmed/9482927. 9482927 10.1073/pnas.95.5.2568 PMC19416

[94] Irazoki O, Mayola A, Campoy S, Barbé J, Henrichsen J, Butler M, et al SOS System Induction Inhibits the Assembly of Chemoreceptor Signaling Clusters in Salmonella enterica. Rao C V., editor. PLoS One. Public Library of Science; 2016;11: e0146685 10.1371/journal.pone.0146685 26784887 PMC4718596

